# Case Study: Intra- and Interpersonal Coherence of Muscle and Brain Activity of Two Coupled Persons during Pushing and Holding Isometric Muscle Action

**DOI:** 10.3390/brainsci12060703

**Published:** 2022-05-29

**Authors:** Laura V. Schaefer, Frank N. Bittmann

**Affiliations:** Devision of Regulative Physiology and Prevention, Department of Sport and Health Sciences, University of Potsdam, Karl-Liebknecht-Str. 24-25, 14476 Potsdam, Germany; bittmann@uni-potsdam.de

**Keywords:** interpersonal muscle action, wavelet coherence, inter-brain synchronization, inter-muscle-brain synchronization, electroencephalography (EEG), mechanomyography (MMG), holding isometric muscle action (HIMA), pushing isometric muscle action (PIMA)

## Abstract

Inter-brain synchronization is primarily investigated during social interactions but had not been examined during coupled muscle action between two persons until now. It was previously shown that mechanical muscle oscillations can develop coherent behavior between two isometrically interacting persons. This case study investigated if inter-brain synchronization appears thereby, and if differences of inter- and intrapersonal muscle and brain coherence exist regarding two different types of isometric muscle action. Electroencephalography (EEG) and mechanomyography/mechanotendography (MMG/MTG) of right elbow extensors were recorded during six fatiguing trials of two coupled isometrically interacting participants (70% MVIC). One partner performed holding and one pushing isometric muscle action (HIMA/PIMA; tasks changed). The wavelet coherence of all signals (EEG, MMG/MTG, force, ACC) were analyzed intra- and interpersonally. The five longest coherence patches in 8–15 Hz and their weighted frequency were compared between real vs. random pairs and between HIMA vs. PIMA. Real vs. random pairs showed significantly higher coherence for intra-muscle, intra-brain, and inter-muscle-brain activity (*p* < 0.001 to 0.019). Inter-brain coherence was significantly higher for real vs. random pairs for EEG of right and central areas and for sub-regions of EEG left (*p* = 0.002 to 0.025). Interpersonal muscle-brain synchronization was significantly higher than intrapersonal one, whereby it was significantly higher for HIMA vs. PIMA. These preliminary findings indicate that inter-brain synchronization can arise during muscular interaction. It is hypothesized both partners merge into one oscillating neuromuscular system. The results reinforce the hypothesis that HIMA is characterized by more complex control strategies than PIMA. The pilot study suggests investigating the topic further to verify these results on a larger sample size. Findings could contribute to the basic understanding of motor control and is relevant for functional diagnostics such as the manual muscle test which is applied in several disciplines, e.g., neurology, physiotherapy.

## 1. Introduction

Muscle fibers oscillate mechanically in frequencies ~10 Hz and can be measured by mechanomyography (MMG) [1,2,3,4,5,6,7]. McAuley postulated that those oscillations reflect the functioning of the neuromuscular system [1]. Intermuscular synchronization in one person is investigated mostly by electromyography (EMG), whereby coherence between two muscles was found, e.g., in frequencies of 8–30 Hz [8,9,10,11,12]. Evidence exists that accelerations (ACC) and EMG can synchronize within one person in low frequency areas [13]. Meanwhile, mutual MMG-EMG recordings are more often used to investigate muscular activity [14,15,16,17,18]. The interaction of muscle and brain activity intrapersonally is usually investigated by EMG and electroencephalography (EEG) or magnetoencephalography (MEG). Coherence between EEG/MEG and EMG was found in frequencies of 8–30 Hz [9,19,20,21,22,23,24,25,26,27,28]. Today, investigations of EEG and MMG are considered [29]. However, to the authors’ knowledge, their coherence has not been considered so far. Furthermore, intermuscular coherence between two persons was not examined until now, except in our own studies [30,31,32]. It was shown that mechanical myotendinous oscillations (measured by MMG and mechanotendography (MTG)) can generate coherent behavior during isometric interaction between two persons with significant differences to randomly matched pairs [30,31,32]. This must be based on complex neuromuscular control mechanisms: not only to control the own muscle fibers of different muscles during isometric action, but also to adjust them to the neuromuscular system of the coupled partner so that a joint muscular rhythm is generated [30,31,32]. Sensorimotor adjustments must be present therefor, which are presumably based on central mechanisms. Since EEG/MEG vs. EMG and EMG vs. ACC/MMG can synchronize intrapersonally during muscular activity [13,19,20,21,22,23,24,26] and since MMG/MTG are able to generate coherent behavior between two interacting persons [30,31,32], it is plausible to also assume that inter-brain synchronization can thereby arise. Studies on that topic are not known. Inter-brain connectivity is rather investigated in social and behavioral sciences [33,34,35,36,37,38,39,40,41,42,43] or joint music performance [44,45,46], e.g., by EEG. Inter-brain synchrony was found in those studies, but with the uncertainty if they are based on ‘real’ inter-brain coupling—which “causally facilitates social interaction” [47,48]—or reflect an epiphenomenon due to perception of the same stimuli [47,48]. According to Hasson and Frith, there are different approaches to interpret the neural coupling (inter-brain synchronization) during social interactions: alignment, conditional transformations, and synergies [49]. The latter refers to the dynamical influence of the activities of both brains to optimize information sharing [49]. This highlights the difficulties of investigating and especially interpreting inter-brain synchronization. However, those investigations are based on social interactions without a muscular coupling between both partners. There seems to be a lack of groundwork regarding the behavior of brain and muscle activity between two persons who are mechanically coupled and muscularly interacting. Since this kind of interaction was not considered before, the question on the quality of a possibly occurring inter-brain synchronization arises. As mentioned above, the interpersonal coherence of muscles was already shown [30,31]. However, muscles are only the executive body. Investigating the patterns of inter-muscle-brain and inter-brain activation will reveal novel basic knowledge on motor control and inter-brain synchronization. These are relevant for sports, movement, and neurosciences and might shed light on the processing of motor control during such a complex interpersonal motor task. Moreover, basic knowledge on the functioning of interpersonal neuromuscular coherence is relevant for diagnostic tools such as the manual muscle test (MMT) applied in different fields, e.g., neurology and physiotherapy, in which examiner and patient get in muscular interaction [50,51,52,53,54].

This preliminary case study aimed to gather first data on the coherence between brain (EEG) and mechanical myotendinous activity (MMG/MTG) intra- and especially interpersonally during isometric muscular interaction of the elbow extensors of two coupled healthy persons. It was hypothesized that intra- and interpersonal coherent behavior of muscle and brain activity is present and differs significantly between real and randomly matched pairs. The latter comparison should address the above-mentioned problem if ‘real’ inter-brain coupling is present or only based on an epiphenomenon. Moreover, two different tasks of isometric muscle action were considered. Several researchers investigated holding and pushing isometric muscle actions (HIMA; PIMA) and differences were found between them [55,56,57,58,59,60,61,62]. It was suggested to differentiate both types and that HIMA is based on more complex control strategies than PIMA [51,52,60,61]. Hence, this pilot investigation might also provide first data on the central processing of HIMA and PIMA during personal muscular interaction. Due to the complexity of the evaluation, only one pair has been evaluated. Based on the small sample size, it must be stated that only preliminary explorative data are provided and that the findings can only be interpreted as first clues on that topic. However, the promising results are considered to be a valuable contribution and, therefore, should be presented as preliminary report.

## 2. Materials and Methods

The objective of this exploratory case study was to collect first data on how mechanical myotendinous oscillations (measured by MMG and MTG; in the following referred to as MMGs) and brain activity (measured by EEG) behave during fatiguing isometric interactions of the elbow extensors of two coupled subjects. Thereby, one partner performed PIMA, whereas the other one executed HIMA (tasks changed). The MMGs and EEGs were measured and analyzed with algorithms of nonlinear dynamics regarding their intra- and interpersonal coherence (wavelet coherence).

### 2.1. Participants

Two healthy, right-handed male students (A and B; study programs related to health and physical activity; University Potsdam, Germany) volunteered to participate in the study. Partner A and B were 28 and 22 years old, weighed 71 kg and 60 kg, and were 178 cm and 173 cm tall, respectively. They reached a maximal voluntary isometric force (MVIC) with their elbow extensors of 186.11 N and 141.97 N (setting see below). Exclusion criteria were complaints of upper extremities, shoulder girdle, and spine, or any other health issue within six months prior to the measurement.

### 2.2. Setting

**Figure 1:**
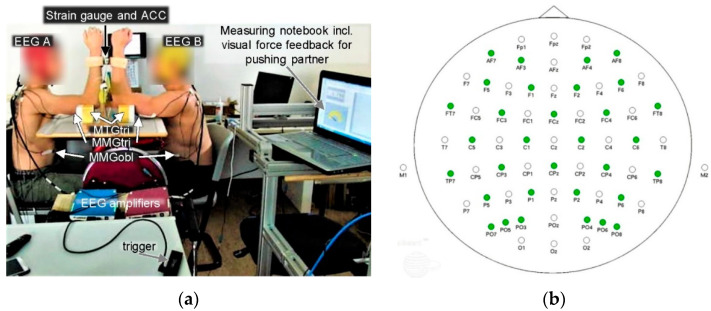
Interpersonal setting. (**a**) Both partners were coupled at their distal forearms and were prepared with electroencephalography (EEG) and mechanomyographic and mechanotendographic sensors (MMG/MTG). A styrofoam functioned as support for the upper arms and included a channel so that the MTG sensors were prevented from a direct contact to the base. An acceleration sensor (ACC) was fixed on the strain gauge. Note: This picture shows a pair of a left and a right-handed participant. The setting of the here measured pair was identical except for the side of performing arms. (**b**) Electrode layout of the 64-channel EEG waveguard™ cap on the scalp. With kind permission of ANTneuro, Hengelo, The Netherlands. (Electrode layout. Available online: https://www.ant-neuro.com/products/waveguard/electrode-layouts (accessed on 1 May 2022)).

The setting (Figure 1a) was related to the one reported in Schaefer and Bittmann [30]. The subjects were sitting opposite but shifted in a way, so that the measured dominant vertically positioned forearms were directly towards each other. The angles between leg and trunk, arm and trunk, as well as the elbow angle measured ~90°. An interface proximal to the ulnar styloid processes connected the subjects. It consisted of two shells of a thermic deformable polymer material shaped according to the contour of forearms. A strain gauge was located between the shells (model: ML MZ 2000 N 36, incl. amplifier; modified by Co. Biovision, Wehrheim, Germany) in order to record and control the reaction force between the subjects. An acceleration sensor (ACC) incl. amplifier (Co. Biovision, Wehrheim, Germany) was fixed on the strain gauge to detect the accelerations along the longitudinal acting force vector.

### 2.3. Mechanomyographic and Mechanotendographic Recordings

The mechanical muscular oscillations of the lateral head of the triceps brachii muscle (MMGtri) and its tendon (MTGtri) as well as the ipsilateral abdominal external oblique muscle (MMGobl) were recorded using a piezoelectric based measurement system. This included pick-ups for clarinets (MMG-sensors; model: Shadow SH 4001, Co. shadow electronics, Erlangen, Germany) and amplifiers for guitars (Nobels preamp booster pre-1, Co. Nobels, Hamburg, Germany), which turned out to be especially suitable to measure MMG and MTG [63]. The piezo-sensors (sensor head) were fixed using tape (usually applied for adhering electrodes of electrocardiography) on the skin above the muscle bellies (greatest protrusion of the muscle during activity in the setting) and above the tendon at the olecranon fossa. Additionally, adhesive tape was used to attach the cable directly behind the sensor head to avoid probably disturbing cable motions. All MMGs, force, and ACC signals were conducted across an analog to digital converter (14-bit; Co. Biovision, Wehrheim, Germany) and were recorded by the software NI DIAdem 10.2 (Co. National Instruments, Austin, TX, USA) on a measurement notebook (Sony Vaio: PCG-61111M, Co. Sony, Tokio, Japan; Windows 7, Co. Microsoft, Redmond, WA, USA). Sampling rate was 1 kHz.

### 2.4. Electroencephalographic Recordings

Two 64-channel EEG-systems (eego™; noise < 1.0 μV rms, resolution 24-bit; Co. ANTneuro, Hengelo, The Netherlands) including a DC amplifier (2 kHz; Co. ANTneuro, Hengelo, The Netherlands) were used to record the EEG of each partner. Waveguard™ original caps (Co. ANTneuro, Hengelo, The Netherlands) with 64 Ag/AgCl electrodes positioned according to the 10/20 international EEG system were fixed on the scalp of the participants (Figure 1b). The ground electrode was CPz. Skin impedances were kept below 10 kΩ and the sampling rate was 1 kHz. The EEG signals were recorded by the eegoTM mylab software package (Co. ANTneuro, Hengelo, The Netherlands). No online pre-processing was applied (data processing see below).

To synchronize the signals recorded with NI DIAdem (MMGs, force, ACC) and the EEG signals, a single button response box was utilized to send a trigger (Figure 1a) to both recording softwares to mark three time points: start of measurement (trigger 1; prior to force application), start (trigger 2), and end (trigger 3) of the isometric plateau.

### 2.5. Measuring Procedure

The measurements took place at a single appointment in the neuromechanics laboratory of the University of Potsdam (Potsdam, Germany). Both participants were introduced to the setting and procedure and gave their written informed consent. Subsequently, EEG, MMG, and MTG sensors were fixed. Afterwards, each participant performed two MVIC measurements separately. For that, they had to push (PIMA) in the later used measurement position against a strain gauge, which was fixed at a stable abutment. The MVIC of the weaker subject (highest value of two trials) was used to calculate the intensity of 70% of the MVIC for the subsequent interpersonal trials. Measurements without motor task followed, one with opened and one with closed eyes. They were executed simultaneously for both subjects in order to control the EEG signals. Then, the PIMA-HIMA trials were performed. Basically, the subjects adjusted an interpersonal isometric muscle action with their forearms at 70% of the MVIC of the weaker subject and maintained this for as long as possible. Six fatiguing trials were performed. The tasks PIMA and HIMA changed alternatingly, whereby partner A started with PIMA and B with HIMA (assigned by coin toss). The partner performing PIMA had to actively generate the force by pushing against the partner’s resistance and control the force level via biofeedback (dial instrument). The holding partner should provide a stable resistance (“wall”) and should just react to the applied force of his partner in an isometric holding manner (HIMA). He received no visual or acoustic feedback. The fatiguing trials ended either if one partner suddenly stopped the resistance (decline in force) or if the forearms deviated more than 7° from the starting position. Three trials in which A performed PIMA and B HIMA (A-PIMA_B-HIMA) as well as three trials in which B performed PIMA and A HIMA (B-PIMA_A-HIMA) were executed in an alternating manner. Resting time between the trials was 120 s. The six fatiguing, the MVIC, and the opened eyes trials were considered for evaluation.

### 2.6. Data Processing

All raw data (EEG, MMGs, force, ACC) of the fatiguing PIMA-HIMA trials were cut from trigger 2 to trigger 3, which refers to the isometric plateau at 70% of the MVIC. The cut EEG raw data were transferred to NI DIAdem to unite the EEG signals with the other ones in one data set for further processing. All signals were checked for quality. The signal-to-noise ratio (SNR) is excellent for MMGs and ACC signals—as always seen by utilizing the above-mentioned measurement system. The unfiltered EEG signals showed a very low SNR, which seems to be usual for EEG [64,65,66]. Therefore, the online filtering is commonly applied. Nevertheless, firstly, the unfiltered EEG signals were visually investigated concerning possible faulty signals, which were never present. Independent component analysis (ICA) was not applied due to the known uncertainty of ICA, which may “influence the underlying EEG signal with a real data set” [67]. Artifacts as eye-blinks were seldomly present only in a few EEG channels. The duration of trials used for coherence analysis was considerably long so that those artifacts would not have a major effect on the outcome of coherence regarding the entire trial. Since eye-blinks did not appear simultaneously between the partners the interpersonal coherence would have been even worse. Moreover, the EEG signals were averaged (see below) and, therefore, the minor occurred artifacts were levelled. The signals of the used MMG measuring system usually do not need pre-processing. Since this investigation considered, inter alia, MMG-EEG coherence, all signals had to be processed identically. Therefore, the common filtering approach for EEG was applied for each signal. Hence, all signals (EEG, MMGs, ACC, force) were filtered using a Notch-Filter (49–51 Hz) and a bandpass filter (Butterworth, Hamming window, window width 25) from 0.016 to 256 Hz according to [68]. Furthermore, the signals were down-sampled from 1000 Hz to 250 Hz. Subsequently, the drift was removed by subtracting the highly filtered signals (Butterworth, filter degree 10, cutoff frequency 1 Hz) from the previously filtered signals. In doing so, the signals were pulled down oscillating around zero. This is necessary for wavelet coherence analysis to avoid leakage effect. Other filtrations were not applied since for wavelet coherence analyses, ideally raw signals should be used. Regarding EEG, there are different partly complex approaches for channel selection depending on the application [69]. Since the present investigation differs clearly from common ones, we decided to basically use an approach suggested by Ernst [70]. She averaged different EEG channels according to 17 anatomical brain regions [70]. We defined ten brain regions (Table 1, named sub-regions in the following). For that, the channel selection was supported by examining the coherence wavelet of each of two different adjacent channels (intrapersonally). Thus, the brain sub-regions were grouped by considering the intensity of coherence of those channels. In case they showed high coherence over the whole duration, they were combined. In case of lower coherence, the channel was excluded from the sub-region and another sub-region was defined. The EEG channels were then averaged according to those ten defined sub-regions (Table 1).

The isometric plateau of the signals of MVIC trials was cut, too. In case it was shorter than 3 s, the starting point was shifted to the force increase, so that at least a duration of 3 s was gained. This is necessary for the wavelet coherence analysis. The data processing was identical to the above-mentioned one. The same applies for the opened eyes trials (OpEy), in which the whole duration was used.

For wavelet coherence analysis, the ten EEG sub-regions, the six MMGs as well as the force and ACC signals were included. Exemplary signals are given as Supplementary Materials (Figure S1).

### 2.7. Wavelet Coherence Analysis

The wavelet coherence analysis was performed using a script programmed in Python (Python Software Foundation, Beaverton, OR, USA), which was compiled in cooperation with the Department of Applied Mathematics, University of Potsdam (Prof. Matthias Holschneider, Dr. Hannes Matuschek) and was used previously [30]. The wavelet coherence enables statements about two non-stationary signals and shows the degree of coherence in specific frequency bands in the course of time [30,31]. This was utilized here to estimate the interaction of the respective signals intra- and interpersonally (EEG sub-regions, MMGs, ACC, force).

The wavelet coherence *Coh_g_*[*s_x_*, *s_y_*] [71] of two time series *s_x_* and *s_y_* was estimated by

$$Coh_{g}\left[s_{x},s_{y}\right]\left(b,a\right)=\frac{CS_{g}\left[s_{x},s_{y}\right]\left(b,a\right)}{\left|\langle W_{g}s_{x}\left(b,a\right)\rangle \right|\left|\langle W_{g}s_{y}\left(b,a\right)\rangle \right|}\;,$$

where *CS* stands for the cross spectrum defined by

$$CS_{g}\left[s_{x},s_{y}\right]\left(b,a\right)=\langle (W_{g}s_{x}\cdot W_{g}s_{y})(b,a)\rangle$$

using the continuous wavelet transformation $\left(W_{g}s\right)\left(b,a\right)=\int_{-\infty }^{\infty }\frac{1}{a}g^{*}\left(\frac{t-b}{a}\right)s\left(t\right)dt$ with the Morlet wavelet as mother wavelet [72]: $g_{\sigma }\left(x\right)=e^{ix}e^{-\frac{x^{2}}{2\sigma^{2}}}$.

The modulus of |*Coh_g_*[*s_x_*, *s_y_*] (*b*, *a*)| ϵ [0, 1] quantifies the coherence of the two time-series [31]. The variance of the cross-wavelet estimator and, therefore, also the coherence wavelet estimator can only be reduced on the cost of increasing bias [73]. In order to separate spurious from significant coherence patterns, a point-wise significance test using surrogate data was implemented in the Python script. A detailed description of the algorithm can be found in Maraun et al. [73]. The frequency borders were defined from 3 to 30 Hz.

For two time series, always one Excel (IBM Microsoft Office, Co. Microsoft, Redmond, WA, USA) and one png file resulted from the wavelet coherence analysis. The Python script bordered the significant coherence patches in the plot (α = 0.05) and extracted the following values in an Excel file: (1) duration of the whole time series (s); (2) number of patches (n); (3) minimal and maximal time points of each patch (s) (refers to the start and the end of each patch); (4) total duration of each patch (s); (5) minimal and maximal frequency of each patch (Hz); and (6) frequency range of each patch (Hz).

### 2.8. Coherence Parameters of Wavelet Coherence Analysis Used for Statistical Evaluation

A second Python script was programmed to extract the following parameters of the Excel files which resulted from the wavelet coherence analysis:

1. Sum5PaD (%): The duration (s) of the five longest significant coherence patches in the frequency range of 8 to 15 Hz were added and this sum was related to the whole duration time (s). Hence, this parameter stands for the ratio (%) of the summed duration of the five longest coherent patches to the total duration time in the respective frequency range. The frequency band of 8–15 Hz was chosen since muscular oscillations are known to be located at ~10 Hz. A value >100% could appear due to the summation of the duration of the five longest significant patches, which might overlap because of different frequencies.
2. WFreq (Hz): This parameter refers to the time-weighted average of the frequency of the five longest significant patches in the frequency range of 8–15 Hz. It should give an impression of the frequencies in which the patches were located. Some considerations included additionally the WFreq of the five longest coherence patches in the frequency range of 3 to 25 Hz.

The frequencies are located in the classical alpha band (~8–14 Hz). However, according to Pfurtscheller and Lopes da Silva [74], we suggest not clearly distinguishing between the bands because clear overlaps arose.

The parameters of wavelet coherence were estimated for each possible intra- and interpersonal signal pair of the EEG sub-regions, MMGs, ACC, and force. In total, 378 signal pairs were considered (force, ACC, 6 × MMGs (3 of partner A, 3 of B), 20 × EEG (10 of A, 10 of B) of each trial (3 × A-PIMA_B-HIMA, 3 × B-PIMA_A-HIMA)).

### 2.9. Statistical Comparisons

Statistical comparisons were, inter alia, performed between real vs. randomly matched signal pairs. This is necessary because randomly matched signal pairs also show significant coherence patches and it is not clear if the patches of real pairs are based on true coherence resulting from the interaction [30]. For the randomly matched signal pairs, the wavelet coherence was also calculated as described above. Thus, two signals were randomly selected out of different measurements. Hence, each possible signal pair (in total 378, see above) was gathered out of different measurements for random pairs.

#### 2.9.1. Real vs. Randomly Matched Pairs

For the statistical comparisons, firstly, the real (AB_IMA) and the randomly matched pairs (rand) were examined regarding possible differences concerning both coherence parameters (Sum5PaD, WFreq) without the consideration of the motor tasks (PIMA vs. HIMA). The applied statistical tests are given below. In general, the EEG sub-regions were combined into three regions: EEG central (EEGcen), EEG left (EEGle), and EEG right (EEGri) for statistics (Table 1). The values for statistical comparisons were obtained as described in the following (concrete examples are given in Appendix A).

*Intrapersonal.* For intrapersonal considerations, the values of Sum5PaD or WFreq of all signal pairs of each partner were considered intrapersonally (MMGs, EEG sub-regions). For that, the values of the respective parameter of one participant of all trials of each configuration (A-PIMA_B-HIMA and B-PIMA_A-HIMA) were averaged. The obtained values of A and B were averaged again (AB_IMA). Hence, all values of A and B regarding one intrapersonal region combination were averaged to receive the respective arithmetic means (M) for statistical comparison (for example, see Appendix A). This was done for each of the ten region combinations (intra-MMGs, intra-EEGcen, intra-EEGle, intra-EEGri, intra-EEGcen-EEGle, intra-EEGcen-EEGri, intra-EEGle-EEGri, intra-MMGs-EEGcen, intra-MMGs-EEGle, and intra-MMGs-EEGri). Additionally, the coefficients of variation (CV) of the averaged values of each region combination were calculated by dividing M by the standard deviation (SD).

For force and ACC signals, the parameters Sum5PaD or WFreq were averaged similarly over the three trials, but separately for A and B. Thus, the values of A and B were not averaged again but considered together in one group because of the otherwise resulting low sample sizes of *n* = 3 or 4.

*Interpersonal.* A similar procedure was applied for interpersonal region combinations. The values of Sum5PaD or WFreq of all interpersonal signal pairs (MMGs, EEG sub-regions) were considered. For each possible region combination (Table A1), the values of the three trials of A-PIMA_B-HIMA and of B-PIMA_A-HIMA were averaged. Subsequently, the M of those two averaged values were calculated (for example, see Appendix A). Those were used for statistical comparisons between real and random pairs classified according to the ten-region combination (inter-MMGs-MMGs, inter-MMGs-EEGcen, inter-MMGs-EEGle, inter-MMGs-EEGri, inter-EEGcen-EEGcen, inter-EEGle-EEGle, inter-EEGri-EEGri, inter-EEGcen-EEGle, inter-EEGcen-EEGri, and inter-EEGle-EEGri). In the following, the coherence parameters of same region comparisons will be named MMGs (=MMGs-MMGs), EEGcen (=EEGcen-EEGcen); analogues for EEGle and EEGri. Additionally, the CVs of the averaged values of each region combination were calculated.

*Further comparisons.* The same procedure was used for the parameters Sum5PaD and WFreq comparing the real AB_IMA vs. MVIC trials, which were performed during single measurements by pushing against a stable resistance (PIMA). Therefore, the intrapersonal coherence of MVIC measurements were based on real comparisons. In contrast, the interpersonal comparisons of MVIC reflect randomly matched trials. They include the same motor task, but without coupling between the partners. This consideration was performed to get an impression of whether inter-brain coherence during real coupled interpersonal measurements differed from non-coupled measurements with motor task.

The Sum5PaD of interpersonal EEG-regions were furthermore compared between the measurements with real isometric muscle interaction (AB_IMA) and the trial with opened eyes without muscular action (OpEy). This was considered because it seems to be conceivable that EEG activity might regularly show coherent phases during any kind of muscular activity. That is why the task of muscular activity should be eliminated for regarding the coherence of interpersonal EEGs.

Normal distribution of all data sets was checked by the Shapiro–Wilk test. The group comparisons between real vs. random (AB_IMA vs. rand), real vs. MVIC, and real vs. OpEy were performed by *t*-test for paired samples for parametric data and by Wilcoxon signed rank test for non-parametric data. The effect size was determined by Cohen’s $d_{z}=\frac{\left|MD\right|}{SD_{MD}}$ for paired *t*-test, where MD is the mean difference of the respective values of each group and *SD_MD_* its standard deviation. The effect sizes were interpreted as small (0.2), moderate (0.5), large (0.80), or very large (1.3) [75,76]. For the Wilcoxon test, the effect size was calculated by $r=\left|\frac{z}{\sqrt{n}}\right|$.

#### 2.9.2. Comparisons between Pushing (PIMA) vs. Holding Isometric Muscle Action (HIMA)

The second objective of the pilot study focused on the investigation of the motor tasks PIMA and HIMA. The parameters Sum5PaD and WFreq were compared between PIMA and HIMA by uniting the data of the trials of both partners, in which the partners performed either PIMA or HIMA. For that, the M of the three trials were calculated for A and B, respectively. For intrapersonal comparisons or for comparisons including ACC or force, the signals of the partner either performing PIMA or HIMA could be clearly distinguished and, therefore, a clear differentiation between HIMA and PIMA was possible. For interpersonal comparisons, a problem arose since in each trial either partner A or B performed PIMA or HIMA, respectively. Hence, each motor task was present concerning the coherence of the signal pairs. To sharpen the comparisons of the data sets, the M of the values of the respective parameters were calculated by the combination of the signal regions of A (or B) towards all signal regions of B (or A) (for a concrete example, see Appendix A).

The data sets of PIMA and HIMA were checked for normal distribution utilizing the Shapiro–Wilk test. In case of normal distribution, a *t*-test for paired samples was executed for interpersonal comparisons; for non-parametric data, the related samples Wilcoxon signed rank test was performed. Effect sizes were calculated as described above. For intrapersonal comparisons, the group of MVIC was included into statistical comparisons since they also reflect a PIMA. Therefore, an ANOVA for repeated measurements (RM ANOVA) was executed. In case, Mauchly’s sphericity was not fulfilled, the Greenhouse Geisser correction (F_Green_) was applied. The effect size of RM ANOVA was given by eta-squared (η^2^).

All statistical comparisons were performed in IBM SPSS Statistics 27 (IBM, Armonk, New York, USA). The significance level was α = 0.05. A large number of comparisons resulted. Due to the explorative character of this preliminary study, we accepted the problem of multiple testing as was suggested by several authors [77,78,79].

## 3. Results

### 3.1. Real vs. Randomly Matched Pairs

#### 3.1.1. Intrapersonal AB_IMA vs. Rand

**Figure 2:**
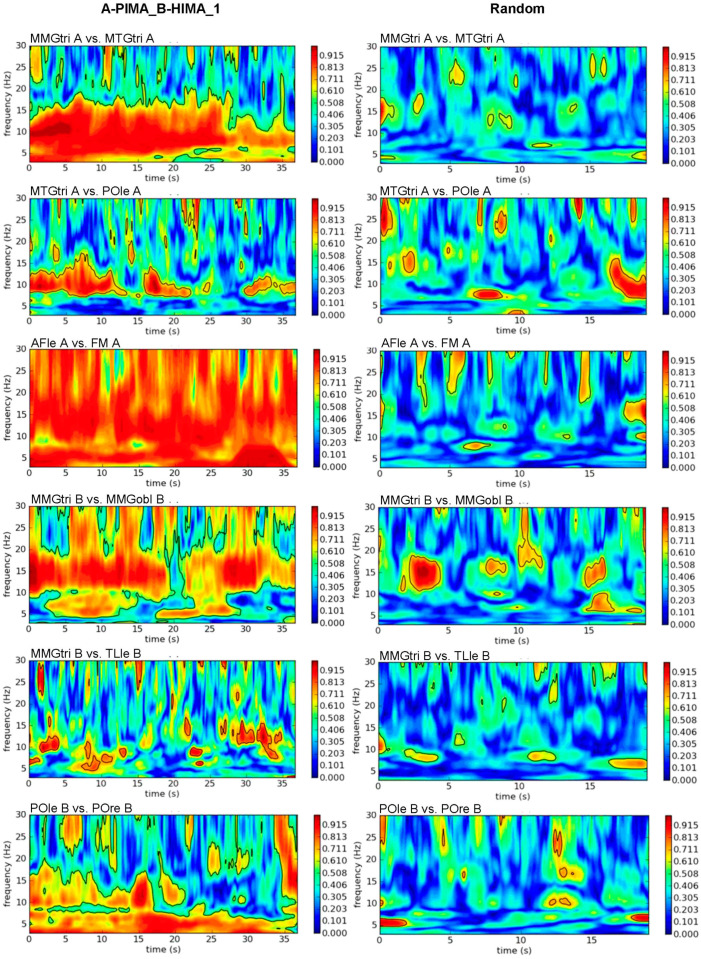
Intrapersonal wavelet coherence. Exemplary plots of wavelet coherence estimation in frequency range of 3 to 30 Hz of selected intrapersonal signal combinations of the first trial of A-PIMA_B-HIMA (**left**) compared to the same signal combinations of randomly matched trials (**right**). The amount of coherence is indicated by the color intensity of the right bar (red = coherence > 0.9).

**Figure 3:**
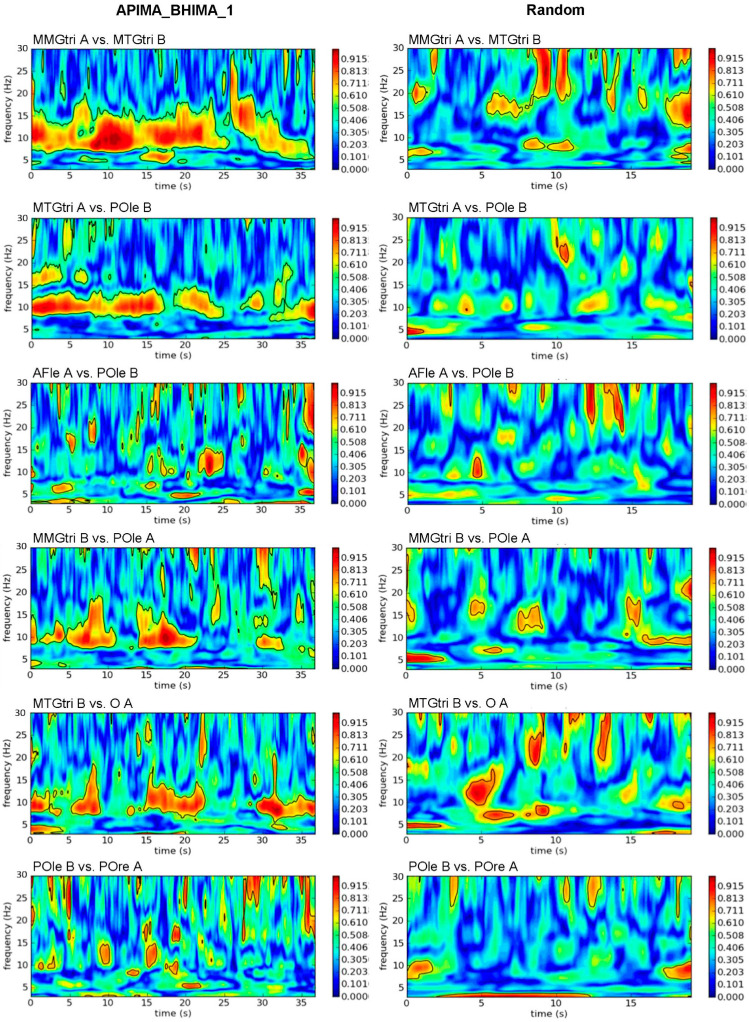
Interpersonal wavelet coherence. Exemplary plots of wavelet coherence estimation in frequency range of 3 to 30 Hz of selected interpersonal signal combinations of the first trial of A-PIMA_B-HIMA (**left**) compared to the same signal combinations of randomly matched trials (**right**). The amount of coherence is indicated by the color intensity of the right bar (red = coherence > 0.9).

As the exemplary plots illustrate (Figure 2), the real in contrast to random signal pairs showed large significant patches with high coherence, except for MMGtri_B-TLle_B. The latter also exhibited a large number of significant patches with high coherence, but they were rather short. The different time axes in Figure 2 and Figure 3 for real and random pairs resulted because evaluating the wavelet coherence for random pairs required the same durations of the trials. Therefore, all trials had to be cut to the shortest measurement (~19 s).

The coherence patches (Sum5PaD) for AB_IMA vs. rand were clearly and significantly longer with very large effect sizes (*p* ≤ 0.001–0.019; *d_z_* = 1.295–10.501) for each region, except for MMGs-EEGcen (*p* = 0.239) and MMG-EEGle (*p* = 0.074) (Table 2). The non-significance is presumably a result of the small sample size (*n* = 3) and an outlier regarding intra-AFle-POle (Sum5PaD = 50.98%). AFle-TLle and TLle-POle showed values of 106.05% and 115.13%, respectively. The CV of Sum5PaD (all regions) was significantly lower for AB_IMA vs. rand (t(9) = −2.887, *p* = 0.018, *d_z_* = 0.913).

The WFreq in 8–15 Hz of intra-muscle-brain coherence was significantly lower for AB_IMA vs. rand regarding MMGs-EEGri with 11.33 ± 0.33 Hz vs. 12.26 ± 0.72 Hz (*p* = 0.006, *d_z_* = 1.250). Regarding 3–25 Hz, the results intensified: MMGs-EEGri and MMGs-EEGle showed significantly lower frequencies for real (~8.62 ± 0.71 Hz) vs. random pairs (~11.72 ± 2.18 Hz) (MMGs-EEGri: *p* = 0.010, *d_z_* = 1.129; MMGs-EEGle: *p* = 0.001, *d_z_* = 1.708). In contrast, the frequencies of intra-brain coherence were significantly higher for real (~12.30 ± 1.35 Hz) vs. random pairs (~9.28 ± 1.88 Hz) (EEGcen: *p* = 0.007, *d_z_* = 1.775; EEGcen-EEGle: *p* = 0.029, *d_z_* = 0.726; EEGle-EEGri: *p* = 0.017, *d_z_* = 1.005).

#### 3.1.2. Interpersonal AB_IMA vs. Rand

The exemplary plots of interpersonal wavelet coherence (Figure 3) show partly clearly lower coherence compared to the intrapersonal comparisons (Figure 2). However, especially inter-MMGs and inter-MMGs-EEG exhibited strong coherence between both partners; inter-brain coherence of real pairs showed only short patches. Statistical comparisons confirmed this (Table 2). Inter-muscle-brain coherence showed significantly higher Sum5PaD for AB_IMA (59.94 ± 4.00%) vs. rand (17.77 ± 3.61%; *p* < 0.001, *d_z_* > 2.31). Coherence of inter-EEGcen and inter-EEGri was significantly higher for real vs. rand with large effect sizes (*p* = 0.011, *d_z_* = 1.003; *p* = 0.025, *d_z_* = 1.291). Inter-EEGle was non-significant comparing AB_IMA vs. rand. Regarding its sub-regions, the same sub-region of both (i.e., AFle_A-AFle_B, TLle_A-TLle_B, and POle_A-POle_B) showed high coherence for random pairs. By excluding them, the real pairs revealed a significantly higher Sum5PaD vs. random (20.35 ± 2.36% vs. 9.58 ± 1.74%; t(2) = 15.363, *p* = 0.002, *d_z_* = 8.870).

The CV of Sum5PaD (all regions) was significantly lower for AB_IMA vs. rand (0.23 ± 0.07 vs. 0.46 ± 0.14; t(9) = −4.489, *p* = 0.002, *d_z_* = 1.420).

Regarding OpEy trials, inter-brain regions showed nearly twice as high Sum5PaD for AB_IMA (20.56 ± 1.07%) vs. OpEy (11.74 ± 1.64%; *p* = 0.001–0.028, *d_z_* > 1.0; Table S1). The CV of Sum5PaD was significantly higher for OpEy (0.45 ± 0.12) vs. AB_IMA (0.20 ± 0.04) (t(5) = −4.564, *p* = 0.006 *d_z_* = 1.863). The WFreq (8–15 Hz) of inter-brain and inter-muscle-brain comparisons did not differ significantly (*p* = 0.097–0.782). The WFreq amounted ~11.57 ± 0.19 Hz for real and 11.52 ± 0.45 Hz for random pairs. The CV of WFreq was significantly higher for rand (0.09 ± 0.02) vs. AB_IMA (0.04 ± 0.02); t(9) = −8.799, *p* < 0.001, *d_z_* = 2.782).

#### 3.1.3. Force and ACC to EEG and MMG: AB_IMA vs. Rand

Sum5PaD of force and ACC to the other regions (MMGs; EEGcen, EEGle, EEGri) and the statistical comparisons between real vs. random pairs are given in Supplementary Material (Table S2). All comparisons were significant. The coherent phases of real MMGs-force and MMGs-ACC lasted mostly over the whole duration indicated by Sum5PaD of ~90% and ~106%, respectively; for random pairs, the respective Sum5PaDs were significantly lower with ~20.18 ± 3.52%. For EEG regions vs. force and vs. ACC, the Sum5PaD of real pairs amounted ~46.45 ± 4.34%. Random pairs revealed still clearly significantly lower Sum5PaD (~19.86 ± 5.75%) with very large effect sizes (*p* = 0.001–0.009; *d_z_* > 1.668). For force-EEGle (*p* = 0.046), force-EEGri (*p* = 0.017), and ACC-EEGri (*p* = 0.024) the significances were not that clear, but large to very large effect sizes were present (*r* = 0.813; *d_z_* > 1.30). Regarding the sub-regions of EEGle, force-POle_A showed a Sum5PaD of 79.98 ± 20.65% for partner A. ACC-POle_A also showed very high coherence (104.25 ± 14.31%). Partner B did not show such high values (force-POle_B: ~39.57 ± 15.78%; ACC-POle_B: ~63.97 ± 23.41%). This exemplifies intraindividual differences of coherence.

The CV of Sum5PaD of force and ACC vs. EEGs and MMGs was generally significantly higher for random (0.70 ± 0.21) vs. real pairs (0.31 ± 0.13; t(7) = −8.111, *p* < 0.001, *d_z_* = 2.868).

The WFreq of all signal pairs of MMGs/EEGs vs. force/ACC amounted ~11.50 ± 0.28 Hz for real and ~11.91 ± 0.45 Hz for random pairs. No significant differences were apparent, except for ACC-EEGri, where real vs. random pairs showed a significantly lower WFreq (11.09 ± 0.50 Hz vs. 12.26 ± 1.32 Hz; t(5) = −3.189, *p* = 0.024, *d_z_* = 1.302). The CVs of WFreq were significantly higher for rand vs. AB_IMA with 0.132 ± 0.06 vs. 0.03 ± 0.01 (t(7) = −4.580, *p* = 0.003, *d_z_* = 1.619).

### 3.2. Coherence of Coupled AB_IMA vs. Single MVIC Trials

#### 3.2.1. Intrapersonal AB_IMA vs. MVIC

During MVIC trials, the MMGs showed −34.25 percent points (pp) lower intrapersonal Sum5PaD vs. coupled AB_IMA, but just missed significance (*p* = 0.065, *d_z_* = 2.146) (Table S3). Intra-brain coherence was also lower for MVIC (79.88 ± 8.75%) vs. AB_IMA (93.63 ± 5.71%), significantly for EEGcen-EEGle (*p* = 0.008, *d_z_* = 0.941) and EEGcen-EEGri (*p* = 0.006, *d_z_* = 0.987) (Table S3). Intra-EEGri just missed significance with *p* = 0.050 (*d_z_* = 2.477). In contrast, for intra-muscle-brain comparisons, Sum5PaD was lower during AB_IMA vs. MVIC trials (29.49 ± 3.87 vs. 43.31 ± 1.92%; n.s.). The CV was significantly lower for AB_IMA vs. MVIC (0.21 ± 0.11 vs. 0.40 ± 0.19; t(9) = −5.297, *p* < 0.001, *d_z_* = 1.675).

#### 3.2.2. Interpersonal AB_IMA vs. MVIC

**Figure 4:**
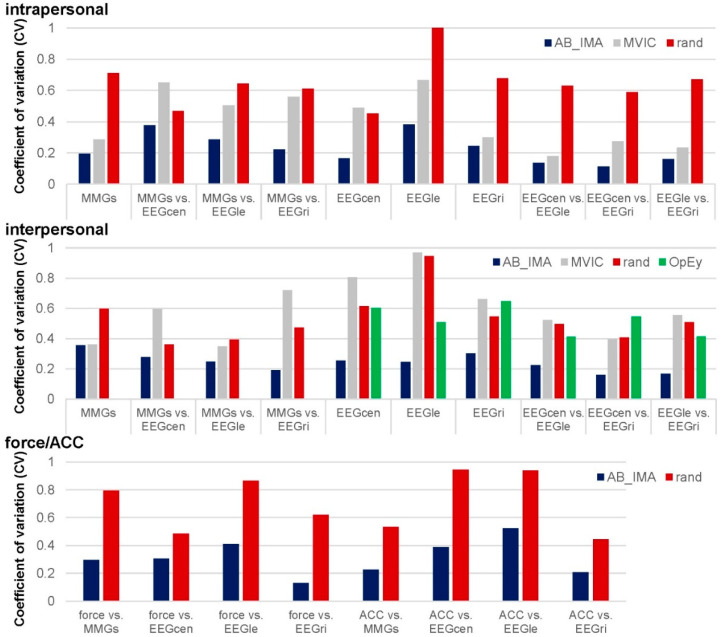
Coefficients of variation of Sum5PaD of all region comparisons intra- and interpersonally. Displayed are the coefficients of variation (CV) of the Sum5PaD of all compared regions regarding intrapersonal (**above**), interpersonal (**middle**) as well as force and ACC (**bottom**) for real coupled pairs (AB_IMA; blue), MVIC trials (gray), randomly matched pairs (rand; red) as well as for the opened eyes trials (OpEy; only regarding interpersonal comparisons; green).

The inter-MMGs comparison revealed a +25.41 pp higher Sum5paD for AB_IMA vs. MVIC (Table S3). The difference was non-significant but clear regarding MMGtri and MTGtri (81.23 ± 11.37% vs. 28.56 ± 15.55%), but including MMGobl, the difference disappeared (43.67 ± 13.58% vs. 45.51 ± 6.70%). The inter-muscle-brain coherence showed a significantly higher Sum5PaD for AB_IMA vs. MVIC (~59.94 ± 4.00% vs. 29.26 ± 2.90%; *p* ≤ 0.001, *d_z_* > 1.61) (Table S3). The CV of inter-muscle-brain Sum5PaD was significantly higher for MVIC vs. AB_IMA (0.55 ± 0.19 vs. 0.24 ± 0.05; t(2) = −8.239, *p* = 0.014, *d_z_* = 4.757). Inter-brain comparisons were non-significant. Regarding MVIC, Sum5PaD of some sub-region combinations were very high, e.g., C_A-FM_B (49.52%), C_A-C_B (42.00%), and C_A-CP_B (68.69%). Some sub-regions revealed low or no coherence, e.g., FM_A-FM_B (0.567%) or CP_A-POc_B (0%). The significantly higher CV of inter-brain Sum5PaD for MVIC vs. AB_IMA highlighted this (0.59 ± 0.13 vs. 0.20 ± 0.04; t(5) = −3.122, *p* = 0.026, *d_z_* = 1.275). Figure 4 displays the CVs of Sum5PaD for all region comparisons and configurations.

### 3.3. Coherence Comparing HIMA vs. PIMA

#### 3.3.1. Intrapersonal HIMA vs. PIMA

The intra-muscle-brain Sum5PaD between HIMA vs. PIMA vs. MVIC were significant for MMGs-EEGri (F_Green_ (1.6, 19.8) = 5.367, *p* = 0.027, η^2^ = 0.24) (Table S4). Pairwise comparisons revealed a significantly higher Sum5PaD for MVIC (43.92 ± 26.25%) vs. PIMA (26.99 ± 7.81%; t(17) = −2.621, *p* = 0.018, *d_z_* = 0.618). Sum5PaD = 30.12 ± 9.55% for HIMA differed not significantly vs. PIMA and vs. MVIC. EEGcen and EEGle vs. MMG were non-significant regarding Sum5PaD comparing HIMA vs. PIMA vs. MVIC. However, Sum5PaD of intra-EEGle-MMGs for partner B was significantly higher during HIMA (41.27 ± 9.88%) vs. PIMA (26.32 ± 6.43%) (t(8) = 5.863, *p* < 0.001, *d_z_* = 1.954). Sum5PaD of intra-EEGri-MMGs of B was close to significance (*p* = 0.079, *d_z_* = 0.671). This did not apply for partner A.

Pairwise comparisons of CV of Sum5PaD (all regions) revealed a significantly higher value for MVIC (0.67 ± 0.07) vs. HIMA (0.34 ± 0.02) (t(2) = 10.777, *p* = 0.009, *d_z_* = 6.222). The CV of PIMA amounted ~0.49 ± 0.19 (n.s.).

#### 3.3.2. Interpersonal HIMA vs. PIMA

**Figure 5:**
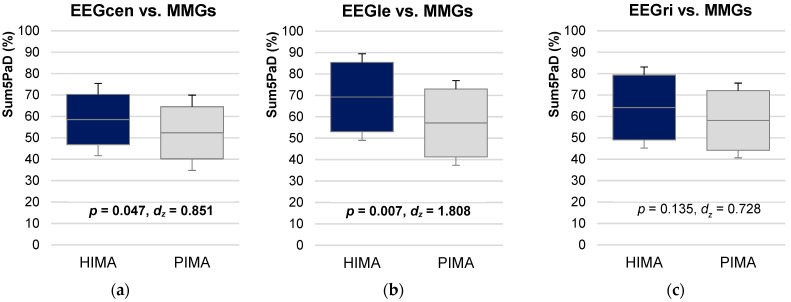
Interpersonal muscle-brain coherence comparing HIMA vs. PIMA. Displayed are the 95%-confidence intervals including arithmetic means and standard deviations (error bars) of the parameter Sum5PaD (**a**) for central areas of EEG vs. MMGs (EEGcen-MMGs), (**b**) for left areas of EEG vs. MMGs (EEGle-MMGs), and (**c**) for right areas of EEG vs. MMGs (EEGri-MMGs) compared between holding isometric muscle action (HIMA, blue) and pushing isometric muscle action (PIMA, gray). The significances p and effect sizes Cohen’s *d_z_* are given.

Inter-muscle and inter-brain Sum5PaD were non-significant comparing HIMA vs. PIMA (*p* = 0.421–1.000). Inter-muscle-brain Sum5PaD was significantly higher for EEGcen-MMGs and EEGle-MMGs during HIMA (58.57 ± 16.87% and 69.24 ± 20.23%, respectively) compared to PIMA (52.38 ± 17.58% and 57.135 ± 19.79%, respectively) (inter-EEGcen-MMGs: t(7) = −2.406, *p* = 0.047, *d_z_* = 0.851; inter-EEGle-MMGs: t(5) = −4.429, *p* = 0.007, *d_z_* = 1.808) (Figure 5, Table S5). EEGri-MMGs showed no significant difference.

The WFreq (8–15 Hz) was significantly lower for inter-EEGri-MMGs during PIMA (11.46 ± 0.58 Hz) vs. HIMA (12.04 ± 0.27 Hz; t(5) = −2.937, *p* = 0.032, *d_z_* = 1.199). The other regions were non-significant (*p* = 0.120–1.000). For 3 to 25 Hz, the WFreq of inter-EEGcen-EEGle (PIMA-HIMA) (8.53 ± 0.63 Hz) was significantly lower than with reverse task (HIMA-PIMA) (9.55 ± 0.56 Hz; t(5) = 11.524, *p* < 0.001, *d_z_* = 4.705). Similar applied for inter-EEGcen-EEGri (PIMA-HIMA: 8.43 ± 0.54 Hz; HIMA-PIMA: 9.53 ± 0.72; t(5) = 2.688, *p* = 0.043, *d_z_* = 1.098). The other comparisons were non-significant.

#### 3.3.3. Force and ACC to All Other Regions: HIMA vs. PIMA

Sum5PaD of force and ACC to the other regions was significantly higher during HIMA vs. PIMA for force-EEGcen (48.95 ± 16.54% vs. 33.81 ± 11.96%; t(7) = −3.108, *p* = 0.017, *d_z_* = 1.099) and force-EEGri (56.06 ± 8.63% vs. 33.44 ± 10.10%; t(5) = −3.758, *p* = 0.013, *d_z_* = 1.534). Force vs. EEG-sub-region AFri showed the highest difference between HIMA and PIMA in both participants (A: 69.29% vs. 25.69%; B: 56.01% vs. 17.81%). The same applied for force-AFle (A: 43.94% vs. 25.99%; B: 52.33% vs. 24.32%). This indicates that during HIMA, the brain of each partner synchronized stronger to the force oscillations than during PIMA.

The WFreq in 8–15 Hz was significantly lower for PIMA vs. HIMA regarding force-EEGle (11.05 ± 0.47 Hz vs. 12.05 ± 0.57 Hz; t(5) = −2.759, *p* = 0.040, *d_z_* = 1.126), force-EEGri (10.96 ± 0.43 Hz vs. 11.96 ± 0.31 Hz; t(5) = −4.578, *p* = 0.005, *d_z_* = 1.943), and ACC-EEGri (10.77 ± 0.48 Hz vs. 11.42 ± 0.60 Hz; t(5) = −3.835, *p* = 0.012, *d_z_* = 1.565). The other comparisons were non-significant.

## 4. Discussion

This preliminary pilot study investigated the wavelet coherence of electrophysiological brain and mechanical muscle activity intra- and interpersonally during muscular interaction of two persons. To the authors’ knowledge, it was the first investigation on this topic. The major objectives were, firstly, to examine if real interpersonal synchronization can basically arise (real vs. random pairs); secondly, if differences between two isometric motor tasks HIMA and PIMA occur. Due to the small sample size, the results have to be interpreted with caution and it is naturally not sure if those will be verified in a larger sample size. Nevertheless, the provided data of this case study should give first hints on the topic of interpersonal muscle-brain-coupling during muscular interaction.

### 4.1. Limitations

The major limitations are the sample size (*n* = 2) and the number of statistical comparisons. Due to the explorative character, we accepted the latter without adjustments according to [77,78,79]. Especially the inter-muscle-brain and intrapersonal coherences showed high significances comparing real vs. random pairs with very large effect sizes so that a multiple testing effect seems not to be likely. However, the large effect sizes are presumably resulting from the small sample size and cannot lead to meaningful conclusions. Therefore, the results can only be interpreted as first indications at this point.

The data processing might show limiting factors. Regarding methodological considerations, the approach of averaging adjacent channels seems to be appropriate. The Sum5PaD and WFreq values were averaged again which resulted in three EEG-regions for statistical comparisons. Thereby, potential effects might have been obscured. The non-existence of clear patterns regarding the coherence of EEG sub-regions (except for above-mentioned ones) might reflect a highly variable inter- and intraindividual EEG expression. Moreover, for MMGs, it might be advisable to separate the MMG of abdominal external oblique muscle from the MMG/MTG of triceps muscle and tendon, since they showed clearly different coherence patterns.

Another limitation has to be mentioned regarding MVIC vs. AB_IMA comparisons, which are based on different force states and intensities, which might have influenced the coherence characteristics.

The findings must be interpreted as preliminary. However, they justify further examinations based on a larger sample size. Some results were so clear and consistent that we interpret them as non-coincidental. However, it is naturally not clear if they will be verified in a larger sample size. Based on the assumption they would be verified in a larger sample size, first neurophysiological consideration on that topic should nevertheless be presented in the subsequent discussion.

### 4.2. Advantages and Disadvantages of Electroencephalography

EEG is a commonly used method for assessing brain activity because of the high temporal resolution, non-invasive, ease of use, and safety [64,65,66]. Disadvantages are a low SNR, low spatial resolution, and the sensitivity regarding muscular activity in the head region as well as concerning heart rate and power line interfaces [64,65,66]. The low spatial resolution is considered as the main disadvantage [80]. The received signal is “the sum of the electric field (in the direction perpendicular to the scalp) that is produced by a large population of neurons” [64]. Therefore, EEG “does not allow researchers to distinguish between activities originating in different but closely adjacent locations” [64]. The EEG is considered to show “spatial blurring” and is regarded as a “low spatial filtering of the cortical potential distribution” [80]. High resolution EEG enhances the spatial resolution [80]. However, due to this limitation of EEG, the interpretation of brain activity in specific locations seems to be difficult. Therefore, the approach of averaging adjacent channels seems to be appropriate.

### 4.3. Corticomuscular Coherence during Coupled Isometric Interaction

Intra- and interpersonal muscle-to-muscle coherence of mechanical oscillations during isometric interaction of two partners was shown previously [30,31,32]. The presented results support those findings: all MMG comparisons between real vs. random pairs differed significantly with very large effect sizes (*d_z_* = 1.5–10.5). The large coherence patches are interpreted as synchronization of the myotendinous oscillations during personal interaction, which can only arise if both neuromuscular systems are able to adapt to each other. This coupling must be controlled by central processes; therefore, a coherence of inter-muscle-brain and inter-brain activity is conceivable. This case study should especially provide a first impression of inter-brain and inter-muscle-brain coherence in such a setting of two muscularly interacting persons. It should again be stated that the discussion has to be interpreted with caution having in mind that only two persons were investigated. However, the results were very clear for inter-muscle-brain coherence comparing real vs. random pairs (*d_z_* > 2.31). This indicated the brain of one partner was able to synchronize to the partner’s mechanical myotendinous oscillations in the sense of coherent behavior. We suggest that this inter-muscle-brain synchronization reflects a specific facet of sensorimotor control during interaction with another oscillatory neuromuscular system. The brain of partner A (or B) is receiving and reacting to the sensorimotor input of partner B (or A). This finding was further supported by the significantly higher coherence of force/ACC vs. EEG for real vs. random pairs (*d_z_* = 1.31–3.68, r = 0.81).

**Figure 6:**
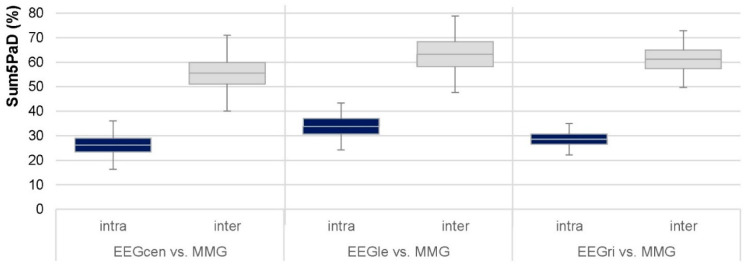
Corticomuscular coherence compared intra- vs. interpersonally. The 95%-confidence intervals including arithmetic means and standard deviations (error bars) of Sum5PaD in the frequency range of 8 to 15 Hz regarding the intrapersonal (blue) and interpersonal (gray) region combinations (AB_IMA) of EEGcen vs. MMGs, EEGle vs. MMGs, and EEGri vs. MMGs are given (*n* = 48, *n* = 36, and *n* = 36, respectively).

Worth highlighting is the significantly higher inter- vs. intrapersonal coherence of corticomuscular activity (Figure 6). The 95%-CIs were clearly disjointed for all regions. This was not expected since the muscle and brain of one person belong to one neuromuscular system. However, the intense coherence between both partners indicates for this case example that both systems can unite to one joint system during interpersonal motor task with a high connectivity between the partners’ muscles and brains. This reflects a higher demand of sensorimotor control for interpersonal than intrapersonal muscle-brain-interaction. The significances and effect sizes of Sum5PaD and its CV between real and random pairs were very clear reflecting a substantial difference, which is interpreted as non-coincidental despite the case study character.

Due to the novel approach, investigations of other researchers do not exist to our knowledge. Some studies considering only one person are related. The beta band activity of brain areas (EEG/MEG) were connected to voluntary motor activity (EMG) [9,19,20,21,22,23,24,25,26,27,28]. The present findings of corticomuscular coherence intra- and especially interpersonally suggest that motor activity is also strongly characterized by lower frequencies (alpha band). Salenius and Hari suggested that a “sensory feedback loop is not necessary for the generation of corticomuscular coherence” [25]. Our results, nevertheless, showed enhanced corticomuscular coherence under the condition of interpersonal interaction. It must be accompanied by intense sensory inputs during the adjustment to the motor action of the counterpart, especially during the holding task, since the participant must react and adapt to the force input of the partner performing PIMA.

We assume the significantly higher inter-muscle-brain vs. intra-muscle-brain coherence might be a result of this sensorimotor regulation and the complex control mechanisms during muscular interaction of two persons, indicating that there is a higher amount of inter-muscle-brain than intra-muscle-brain coordination during personal interaction. The joint rhythm can only arise with a kind of clock generator, which has to be located in central structures. The olivocerebellar circuitry was suggested to undertake a decisive role in temporal-spatial processing, whereby the cerebellum is considered as the most relevant sensorimotor structure [81,82,83,84,85]. Furthermore, the supplementary motor area and the premotor cortex are involved in temporal processing of motor activity [86,87,88]. We assume that other central structures, such as the thalamus, the cingulate cortex, and the basal ganglia [85,89,90,91,92,93,94,95,96,97], are participating during the execution of such a complex interpersonal motor task. Intrapersonal corticomuscular coherence already has to be based on complex control processes; an interpersonal one must entail even higher regulatory demands. The ability of both neuromuscular systems to generate a mutual rhythm of mechanical muscle and electrophysiological brain oscillations in this case reflects the tremendous capacity of neuromuscular systems regarding their dynamic adaptability. Such interactions actually require two properly functioning regulatory systems. In turn, it seems to be conceivable that such a fragile oscillating dynamic equilibrium could easily be interfered with by impairing influences. It was previously shown that during muscular interaction in the sense of the Adaptive Force (AF) assessed by a manual muscle test (MMT), which tests the holding capacity of a person, mutual oscillations appear in stable neuromuscular systems, whereas in impaired ones, oscillations are missing [51,52,53]. This might reflect the oscillatory coherence in undisturbed interacting neuromuscular systems. During MMT, the participant has to adapt in an isometric holding manner to an external increasing force application of the examiner (PIMA) [50,51,52]. Hence, a similar task exists compared to the here presented one. Gaining information on brain activity during such muscular interactions between two persons might help understanding the underlying neuromuscular control processes in case of an impaired holding capacity. Furthermore, Parkinson patients showed altered patterns of mechanical muscle oscillations already in premotor stages, especially by pushing the hands against each other, thus interacting with oneself [98,99]. It can only be assumed how a personal muscular interaction would be characterized in such cases.

### 4.4. Comparison of Holding and Pushing Isometric Motor Tasks (HIMA vs. PIMA)

Regarding intrapersonal coherence, only partner B showed significantly higher coherence during HIMA vs. PIMA for MMG-EEGle (*d_z_* = 1.95), MMG-EEGri was close to significance (*p* = 0.079). If there are generally intrapersonal differences or if this might be a sign that partner B executed the motor tasks in a better way than A remains open. It could still reflect an incidental finding but is assumed to be an actual effect because of the high effect size and the appearance of the finding regarding EEGle. EEGle should reflect the motor task with the right arm more pronounced than EEGri. Regarding the sub-regions, it was visible that Sum5PaD of intra-AFle-POle was higher during HIMA vs. PIMA in both participants (63.08 ± 10.98% vs. 38.88 ± 17.00%). If this would hold true in a larger sample size, it could indicate that HIMA needs a higher amount of synchronization in specific brain areas.

The inter-muscle-brain synchronization (MMG-EEGcen; MMG-EEGle) was significantly higher during HIMA vs. PIMA. MMG-EEGri showed no significant difference between both tasks. The highest significance was present for MMGs-EEGle, which again might reflect the motor task performance with the right arm [100]. Nevertheless, EEGri seems to occupy a special role during this personal interaction due to the higher inter-brain coherence comparing real vs. random pairs. Still, this might not characterize the HIMA-PIMA tasks, but the interpersonal muscle action in general.

The higher coherence for inter-muscle-brain during HIMA vs. PIMA, but not for inter-brain or intra-muscle-brain in this case example might indicate that HIMA probably requires higher sensorimotor control processes between the brain of one partner and the muscles of the other one during such a coupled motor task. During PIMA, the participants initiated the force application but did not have to react as intensely to the partner’s input as during HIMA. It was hypothesized previously that HIMA might involve control strategies related to eccentric muscle action and PIMA rather those of concentric contractions [60,61,62]. The higher requirements for motor control processes during eccentric muscle action are secured [101,102,103,104]. The presented findings of a higher inter-muscle-brain coherence might support the hypothesis that control strategies during HIMA are more complex and, therefore, are probably related to neural processes during eccentric actions. That HIMA might be controlled by more complex neuronal control processes is furthermore supported by findings concerning the AF. The execution of AF is based on HIMA in reaction to a varying external load. It reflects the adaptive holding capacity of the neuromuscular system. In previous studies, the AF was assessed by the above-mentioned MMT. The maximal isometric AF was reduced by perceiving negative stimuli as unpleasant food imaginations or odors and, hence, was interpreted to be more vulnerable than PIMA [51,52,53]. This might reflect the more complex control circuitries in central structures during HIMA vs. PIMA, in which other inputs are also processed, e.g., emotions. It is known that central structures processing emotions are also relevant for motor control [85,90,91,96,105,106] and, hence, emotions can influence the motor output [96]. HIMA might be especially suitable to investigate the effect of negative stimuli (e.g., emotions, nociception) on the motor output. The higher coherence of inter-muscle-brain coherence during HIMA vs. PIMA in this case example might be a first neuroscientific hint for a more complex adjustment of muscle and brain activity during holding actions.

The significantly lower WFreq in 3–25 Hz of inter-brain coherence (EEGle-EEGcen, EEGri-EEGcen) during HIMA vs. PIMA (*d_z_* = 4.71, *d_z_* = 1.10) might also reflect further possible differences between both motor tasks for inter-brain synchronization. It was not expected to find significant differences regarding the frequency between HIMA and PIMA since they were missing in previous studies regarding muscular activity. The amplitude variation, frequency, and power distribution rather showed differences [58,60,61]. Investigating those parameters for EEG could lead to further insights regarding both motor tasks. However, the frequency might be an important parameter investigating inter-brain synchronization comparing HIMA vs. PIMA. Indeed, the findings of this case study are not appropriate to make any conclusions on this topic, but they might point out that it could be worthful to include the frequency consideration in further examinations.

### 4.5. Inter-Brain Synchronization as an Epiphenomenon?

As mentioned in the introduction, inter-brain synchronization is especially investigated during joint guitar playing [44,45,46] or social interactions [33,34,35,49]. During joint guitar playing, each subject perceives the same acoustic input [44,46,107]. Inter-brain couplings were also present if one participant played guitar and the other one listened, indicating the acoustic input already provokes an inter-brain synchronization [46]. Furthermore, proprioceptive and tactile inputs as well as motor action happen simultaneously during guitar playing, which could trigger mutual EEG patterns. Therefore, the reasonable criticism arose if inter-brain synchronization in such settings just occurs due to the perception of the same stimuli and, hence, reflects an epiphenomenon [47,48]. From our point of view, the here performed comparison of real vs. random pairs could be useful to investigate whether or not inter-brain synchronization is related to an epiphenomenon.

Although logically conceivable that due to the remarkable inter-muscle-brain coherence, inter-brain synchronization would also be present, the results were not as clear as expected. The inter-EEGcen and inter-EEGri of both partners showed significantly higher coherence comparing real vs. random pairs (*d_z_* = 1.00–1.29); however, inter-EEGle did not show this behavior. The coherence of same sub-regions of both partners (AFle_A-AFle_B, TLle_A-TLle_B and POle_A-POle_B) was also high for random pairs. By excluding them, the interpersonal coherence of EEGle was significantly higher for real vs. random pairs with a very large effect size (*d_z_* = 8.87). Thereby, the Sum5PaD for random pairs was similarly low as for non-coupled non-motor tasks. Hence, for this case example, inter-brain synchronization seems not only to be based on an epiphenomenon resulting from the performed motor task; specific brain regions could reflect ‘real’ inter-brain synchronization which is assumed to be based on the synergy type of neuronal coupling according to Hasson and Frith [49]. The partly high coherence for random pairs might have occurred since random pairs were taken from the real coupled trials only by matching different measurements. It is assumed that central activities during isometric tasks are generally similar. This is supported by the significantly lower inter-brain-coherence of uncoupled non-motor (OpEy) vs. real pairs for all brain regions (*d_z_* = 1.01–2.05, *r* > 0.78). This indicates that without a motor task or any other interaction, the partners’ brains show rather spurious Sum5PaD of ~12%. The findings of significantly higher inter-EEGri and inter-EEGcen coherence in real pairs rather speaks for a ‘real’ inter-brain synchronization for this case example. On the one hand, it could still be a random finding due to multiple testing and the low sample size; on the other hand, it cannot be ruled out that central and right brain areas might undertake specific functions in the present complex interpersonal motor task. Since the right arms executed the motor action, a stronger inter-brain synchronization was expected for left areas. However, the significant presence of inter-brain-coherence for EEGri and EEGcen in this case example might reveal first hints that those brain areas occupy a specific function during the regulation and control of complex interpersonal motor tasks. The EEGle might rather represent the general execution of the sensorimotor task with the right arm, which is supported by the high inter-MMG-EEGle coherence. This could explain why the random pairs also showed a considerably high interpersonal coherence for EEGle, especially regarding same sub-regions. Another parameter speaking for a real inter-brain synchronization is the significantly lower CV in real vs. random pairs (*d_z_* = 1.75). This points out that the coherence seems to be more consistent for real than random pairs at least in this case example.

It is concluded that a mixture of real inter-brain connectivity and the appearance of an epiphenomenon was present regarding the here investigated pair of participants. An external stimulus, such as the acoustic one mentioned above, did not exist here, but there were proprioceptive and tactile inputs from the counterpart and, of course, both partners were muscularly active. However, the significant differences of Sum5PaD for inter-EEGri, inter-EEGcen, and for some sub-regions of inter-EEGle as well as the differences in CV might be interpreted as possible signs for ‘real’ inter-brain coupling rather than an epiphenomenon during muscular interaction at least regarding the two participants of this case example.

## 5. Conclusions

To our knowledge, the presented case study was the first investigating inter-brain and inter-muscle-brain synchronization during a coupled isometric motor task. Having in mind that only one pair of two interacting participants was investigated, the findings can only be considered as preliminary, providing first hints on the topic. If they will be verified in a larger sample size remain open. Consistent with previous findings, an inter-muscle synchronization was present indicating that both neuromuscular systems were able to agree to a mutual rhythm. The novel finding was that an inter-muscle-brain synchronization arose between both participants which differed significantly from random pairs for all brain regions. The inter-brain synchronization was not that clear, however, showing significant differences to random pairs regarding right and central brain areas and also for sub-regions of the left hemisphere. Furthermore, the CVs were significantly lower for real vs. random pairs. It is hypothesized therefore that inter-brain synchronization might be partly based on ‘real’ synchronization and partly on an epiphenomenon due to the motor action. This indicates, at least for this pair of participants, that their neuromuscular systems were not only able to adjust their own activities between muscles and/or brain intrapersonally, but also that it is in principle possible that a neuromuscular system is able to adjust and synchronize to another coupled neuromuscular system in low frequency areas. Due to the found lower intra- than interpersonal muscle-brain coherence, it is assumed that the systems of both partners merge into one united neuromuscular system during muscular interaction. Thereby, the brain of the holding partner seems to couple more strongly to the muscular oscillations of the partner than to the own ones. This could be a possible first hint that during HIMA, the brain probably processes more complex information than during PIMA. It is assumed that this might be the results from the reaction and adaptation during HIMA to the force input of the partner. A higher involvement of the somatosensory areas can be expected by this. Hence, higher requirements regarding control processes are presumed for HIMA vs. PIMA, which supports the current hypothesis.

The findings can only be considered as preliminary results since only one couple was investigated. Since some results appeared consistently and clear, we assume that it is unlikely those are related to incidental findings. At least the results justify further examinations, which will show if the inter-brain synchronization is based on random effects or on true connectivity. The next step will be to investigate the topic on a larger sample size.

These preliminary findings might provide first novel indications on motor control during a complex task of interpersonal muscular actions, which could be relevant for sport and training sciences, kinesiology, and neurosciences. It could also be of interest for functional diagnostic approaches as the manual muscle test measured by the Adaptive Force. This adaptive holding capacity, which is based on HIMA, was recently suggested to be especially vulnerable to interfering stimuli, which might probably be explained by the required high complex control processes.

## Figures and Tables

**Table 1:** EEG regions and sub-regions. Overview of the ten defined EEG sub-regions, related abbreviations, and EEG channels. CPz was set as reference electrode and, therefore, is missing here. For statistical purposes, the coherence parameters (see below) of sub-regions were again averaged, which led to three regions: EEG central (EEGcen), EEG left (EEGle), and EEG right (EEGri) (see statistics).

No.	Sub-Regions	Abbr.	EEG Channels	Regions for Statistics
1	Frontal central	FM	Fpz, Fz	EEGcen
2	Central	C	FC1, FCz, FC2, C1, Cz, C2; CP	
3	Central parietal	CP	CP1, CP2	
4	Parietal occipital central	POc	Pz, POz, Oz	
5	Anterior frontal left	AFle	Fp1, AF3, AF7, F1, F3, F5, F7	EEGle
6	Temporal lateral left	TLle	FC3, FC5, FT7, C3, C5, T7, CP3, CP5, TP7, M1	
7	Parietal occipital left	POle	P1, P3, P5, P7, PO3, PO5, PO7, O1	
8	Anterior frontal right	AFri	Fp2, AF4, AF8, F2, F4, F6, F8	EEGri
9	Temporal lateral right	TLri	FC4, FC6, FT8, C4, C6, T8, CP4, CP6, TP8, M2	
10	Parietal occipital right	POri	P2, P4, P6, P8, PO4, PO6, PO8, O2	

**Table 2:** Intra- and interpersonal Sum5PaD between real and randomly matched trials. Arithmetic means (M), standard deviations (SD), coefficients of variation (CV), *t* values of paired *t*-test or z-value of Wilcoxon test, degrees of freedom (df), significances (p), and effect sizes Cohen’s *d_z_* of all region comparisons intrapersonally (above) and interpersonally (below) regarding the five longest coherence patches related to the whole duration (Sum5PaD (%)) for real isometric muscle interaction (AB_IMA) vs. random pairs (rand) are given. (MMGs: mechanomyography/mechanotendography; EEGcen: Electroencephalography (EEG) of central areas; EEGle: EEG of left areas; EEGri: EEG of right areas).

Sensor Pair	Mode	*n*	M (%)	SD	CV	t/z	df	*p*	*d_z_*
**Intrapersonal**									
MMGs	AB_IMA	3	97.097	4.895	0.050	18.189	2	**0.003**	10.501
	rand		11.982	3.356	0.280				
MMGs vs. EEGcen	AB_IMA	12	26.164	9.840	0.376	−1.177	-	0.239 *	-
	rand		21.864	7.744	0.354				
MMGs vs. EEGle	AB_IMA	9	33.735	9.623	0.285	4.778	8	**0.001**	1.593
	rand		20.95	10.549	0.504				
MMGs vs. EEGri	AB_IMA	9	28.557	6.406	0.224	3.886	8	**0.005**	1.295
	rand		17.190	8.995	0.523				
EEGcen	AB_IMA	6	89.240	14.730	0.165	11.136	5	**<0.001**	4.547
	rand		20.966	5.770	0.275				
EEGle	AB_IMA	3	90.717	34.711	0.383	3.467	2	0.074	2.002
	rand		27.188	3.027	0.111				
EEGri	AB_IMA	3	99.323	24.465	0.246	7.059	2	**0.019**	4.076
	rand		21.312	12.492	0.586				
EEGcen vs. EEGle	AB_IMA	12	90.205	12.243	0.136	15.783	11	**<0.001**	4.556
	rand		22.513	8.841	0.393				
EEGcen vs. EEGri	AB_IMA	12	89.883	10.240	0.114	18.176	11	**<0.001**	5.247
	rand		16.879	6.259	0.371				
EEGle vs. EEGri	AB_IMA	9	102.419	16.432	0.160	19.358	8	**<0.001**	6.453
	rand		16.718	7.804	0.467				
**Interpersonal**									
MMGs	AB_IMA	6	62.450	23.429	0.375	3.636	5	**0.015**	1.484
	rand		18.658	7.535	0.404				
MMGs vs. EEGcen	AB_IMA	12	55.472	15.429	0.278	8.029	11	**<0.001**	2.318
	rand		21.788	7.855	0.361				
MMGs vs. EEGle	AB_IMA	9	63.187	15.588	0.247	9.182	8	**<0.001**	3.061
	rand		16.734	6.579	0.393				
MMGs vs. EEGri	AB_IMA	9	61.155	11.564	0.189	8.762	8	**<0.001**	2.921
	rand		14.797	6.993	0.473				
EEGcen	AB_IMA	10	19.082	4.678	0.245	3.17	9	**0.011**	1.003
	rand		16.412	5.941	0.362				
EEGle	AB_IMA	6	21.94	4.974	0.227	−0.672	5	0.531	0.274
	rand		27.239	22.442	0.824				
EEGri	AB_IMA	6	20.556	3.481	0.169	3.163	5	**0.025**	1.291
	rand		12.783	4.473	0.350				
EEGcen vs. EEGle	AB_IMA	12	20.874	4.645	0.223	0.238	11	0.817	0.069
	rand		20.043	9.989	0.498				
EEGcen vs. EEGri	AB_IMA	12	19.603	3.126	0.159	−0.538	11	0.602	0.155
	rand		20.823	8.474	0.407				
EEGle vs. EEGri	AB_IMA	9	21.320	3.573	0.168	0.501	8	0.630	0.167
	rand		19.534	9.913	0.507				

* Wilcoxon test. Significant results are written in bold.

## Data Availability

Data are contained within the article and the Supplementary Material. Further data (single values of each region and participant) are available on request from the corresponding author.

## Supplementary Materials

The following supporting information can be downloaded at: https://www.mdpi.com/article/10.3390/brainsci12060703/s1, Figure S1: Exemplary signals; Table S1: Inter-brain Sum5PaD motor vs. non-motor tasks; Table S2: Sum5PaD of force and ACC to MMGs and EEG real vs. random pairs; Table S3: Intra- and interpersonal Sum5PaD of coupled real vs. single-MVIC pairs; Table S4: Intrapersonal muscle-brain coherence HIMA vs. PIMA; Table S5: Interpersonal muscle-brain coherence HIMA vs. PIMA.

## Appendix A. Examples for Statistical Data Preparation for Sum5PaD and WFreq

*Intrapersonal.* Example for region combination MMGs-EEGri (MMGs consists of sub-regions MMGtri, MTGtri and MMGobl for A and B; EEGri consists of sub-regions AFri, TLri and POri for A and B): The arithmetic mean (M) of the values of Sum5PaD (or WFreq) of the three trials of partner A regarding the configuration A-PIMA_B-HIMA was calculated for each sub-region: M of three trials of MMGtri_A vs. AFri_A, of MMGtri_A vs. TLri_A, of MMGtri_A vs. POri_A, of MTGtri_A vs. AFri_A, of MTGtri_A vs. TLri_A, of MTGtri_A vs. POri_A and of MMGobl_A vs. AFri_A, of MMGobl_A vs. TLri_A and of the three trials of MMGobl_A vs. POri_A. The same was performed for the values of A regarding the configuration B-PIMA_A-PIMA and for the values of B for both configurations. Thus, for each sub-region, four arithmetic means (related to each configuration) were obtained. Those were averaged again (AB_IMA). Hence, all values of A and B regarding one intrapersonal region combination (MMGs-EEGri) were averaged to receive the respective values for the statistical comparison. This was done for each of the ten region combinations (intra-MMGs, intra-EEGcen, intra-EEGle, intra-EEGri, intra-EEGcen-EEGle, intra-EEGcen-EEGri, intra-EEGle-EEGri, intra-MMGs-EEGcen, intra-MMGs-EEGle, and intra-MMGs-EEGri).

*Interpersonal.* Example for region combination MMGs-EEGle (MMGs consists of sub-regions MMGtri, MTGtri and MMGobl for A and B; EEGle consists of sub-regions AFle, TLle and POle for A and B): Firstly, the M of the values of the three trials during A-PIMA_B-HIMA was calculated for each interpersonal signal pair (MMGtri_A vs. AFle_B, MMGtri_A vs. TLle_B, MMGtri_A vs. POle_B; MTGtri_A vs. AFle_B, MTGtri_A vs. TLle_B, MTGtri_A vs. POle_B; MMGobl_A vs. AFle_B, MMGobl_A vs. TL_B, MMGobl_A vs. POle_B). Thus, nine averaged values resulted. The same was performed for the signal pairs of B vs. A still during A-PIMA_B-HIMA (thus, MMGtri_B vs. AFle_A, etc.) as well as for the configurations A vs. B and B vs. A for the three B-PIMA_A-HIMA trials. Thus, nine averaged values resulted for each of the four configurations (A vs. B and B vs. A of A-PIMA_B-HIMA and B-PIMA_A-HIMA). They were averaged again for each sub-region (exemplified by the colored “x” for MMGs-EEGle in Table A1; the values (x) of the same color were averaged to unite the values of A and B (AB_IMA)). Subsequently, the resulting nine values of each configuration were averaged, so that one value resulted per region combination despite of the configuration and sub-region. Those were used for statistical comparisons between real and random pairs classified according to the ten-region combination (inter-MMGs-MMGs, inter-MMGs-EEGcen, inter-MMG-EEGle, inter-MMGs-EEGri, inter-EEGcen, inter-EEGle, inter-EEGri, inter-EEGcen-EEGle, inter-EEGcen-EEGri, inter-EEGle-EEGri).

*Interpersonal HIMA* vs. *PIMA*. Example regarding EEGle-MMGs: The averaged values over the three trials as explained above were taken as basis. They were averaged by calculating the M coming from the EEG sub-region of the PIMA or HIMA performing partner: For PIMA, the following six values of Sum5PaD or WFreq were averaged: AFle_A vs. MMGtri_B, vs. MTGtri_B and vs. MMGobl_B of the configuration in which A performed PIMA (A-PIMA_B-HIMA) and AFle_B vs. MMGtri_A, vs. MTGtri_A and vs. MMGobl_A of the configuration in which B performed PIMA (B-PIMA_A-HIMA). Those six averaged values were averaged again to obtain the value of PIMA for EEGle-MMGs.

For HIMA, it was the same procedure but the used configurations changed vice versa. The following six values were averaged: AFle_A vs. MMGtri_B, vs. MTGtri_B, vs. MMGobl_B of the configuration B-PIMA_A-HIMA and AFle_B vs. MMGtri_A, vs. MTGtri_A, vs. MMGobl_A of the configuration A-PIMA_B-HIMA, respectively. Those six averaged values were averaged again to obtain the value of HIMA for EEGle-MMGs.

Thus, the averaged values were obtained coming from the EEG sub-regions of the partner who either performed PIMA or HIMA. The same applies for EEGri vs. MMGs; for EEGcen vs. MMGs eight arithmetic means resulted, since EEGcen contains four sub-regions.

**Table A1:** Interpersonal comparisons of all signal pairs. Comparisons were done for MMGs of A vs. MMGs of B (black bordered), EEGcen of A vs. EEGcen of B (light gray), EEGle of A vs. EEGle of B (dark gray), EEGri of A vs. EEGri of B (light gray), MMGs vs. EEGcen (A vs. B and B vs. A; averaged; AB_IMA; blue), MMGs vs. EEGle (AB_IMA, yellow), MMGs vs. EEGri (AB_IMA, green), EEGcen vs. EEGle (AB_IMA, red), EEGle vs. EEGri (AB_IMA, orange).

## References

[1] McAuley J.H. (2000). Physiological and Pathological Tremors and Rhythmic Central Motor Control. Brain.

[2] Beck T. (2010). Applications of Mechanomyography for Examining Muscle Function.

[3] Orizio C., Perini R., Diemont B., Maranzana Figini M., Veicsteinas A. (1990). Spectral Analysis of Muscular Sound during Isometric Contraction of Biceps Brachii. J. Appl. Physiol..

[4] Barry D.T. (1987). Acoustic Signals from Frog Skeletal Muscle. Biophys. J..

[5] Barry D.T., Cole N.M. (1988). Fluid Mechanics of Muscle Vibrations. Biophys. J..

[6] Cescon C., Madeleine P., Farina D. (2008). Longitudinal and Transverse Propagation of Surface Mechanomyographic Waves Generated by Single Motor Unit Activity. Med. Biol. Eng. Comput..

[7] Frangioni J.V., Kwan-Gett T.S., Dobrunz L.E., McMahon T.A. (1987). The Mechanism of Low-Frequency Sound Production in Muscle. Biophys. J..

[8] Laine C.M., Valero-Cuevas F.J. (2017). Intermuscular Coherence Reflects Functional Coordination. J. Neurophysiol..

[9] Farmer S.F., Bremner F.D., Halliday D.M., Rosenberg J.R., Stephens J.A. (1993). The Frequency Content of Common Synaptic Inputs to Motoneurones Studied during Voluntary Isometric Contraction in Man. J. Physiol..

[10] Kilner J.M., Baker S.N., Salenius S., Jousmäki V., Hari R., Lemon R.N. (1999). Task-dependent Modulation of 15–30 Hz Coherence between Rectified EMGs from Human Hand and Forearm Muscles. J. Physiol..

[11] Ishii T., Narita N., Endo H. (2016). Evaluation of Jaw and Neck Muscle Activities While Chewing Using EMG-EMG Transfer Function and EMG-EMG Coherence Function Analyses in Healthy Subjects. Physiol. Behav..

[12] Wang L., Lu A., Zhang S., Niu W., Zheng F., Gong M. (2015). Fatigue-Related Electromyographic Coherence and Phase Synchronization Analysis between Antagonistic Elbow Muscles. Exp. Brain Res..

[13] Coates M., Baker S., Fitzgerald W. (1997). A Complex Wavelet-Transform Approach to Power and Coherence Measurement from Non-Stationary Data. J. Physiol..

[14] Hill E.C., Housh T.J., Smith C.M., Cochrane K.C., Jenkins N.D.M., Schmidt R.J., Johnson G.O. (2017). The Effects of Work-to-Rest Ratios on Torque, Electromyographic, and Mechanomyographic Responses to Fatiguing Workbouts. Int. J. Exerc. Sci..

[15] Yoshitake Y., Ue H., Miyazaki M., Moritani T. (2001). Assessment of Lower-Back Muscle Fatigue Using Electromyography, Mechanomyography, and near-Infrared Spectroscopy. Eur. J. Appl. Physiol..

[16] Tarata M.T. (2003). Mechanomyography versus Electromyography, in Monitoring the Muscular Fatigue. BioMed. Eng. OnLine.

[17] Camic C.L., Housh T.J., Zuniga J.M., Bergstrom H.C., Schmidt R.J., Johnson G.O. (2014). Mechanomyographic and Electromyographic Responses During Fatiguing Eccentric Muscle Actions of the Leg Extensors. J. Appl. Biomech..

[18] Kassolik K., Jaskólska A., Kisiel-Sajewicz K., Marusiak J., Kawczyński A., Jaskólski A. (2009). Tensegrity Principle in Massage Demonstrated by Electro- and Mechanomyography. J. Bodyw. Mov. Ther..

[19] Conway B.A., Biswas P., Halliday D.M., Farmer S.F., Rosenberg J.R. (1997). Task-Dependent Changes in Rhythmic Motor Output during Voluntary Elbow Movement in Man. J. Physiol..

[20] Tass P., Rosenblum M.G., Weule J., Kurths J., Pikovsky A., Volkmann J., Schnitzler A., Freund H.-J. (1998). Detection of N: M Phase Locking from Noisy Data: Application to Magnetoencephalography. Phys. Rev. Lett..

[21] Halliday D.M., Conway B.A., Farmer S.F., Rosenberg J.R. (1998). Using Electroencephalography to Study Functional Coupling between Cortical Activity and Electromyograms during Voluntary Contractions in Humans. Neurosci. Lett..

[22] Farmer S.F. (1998). Rhythmicity, Synchronization and Binding in Human and Primate Motor Systems. J. Physiol..

[23] Conway B.A., Halliday D.M., Farmer S.F., Shahani U., Maas P., Weir A.I., Rosenberg J.R. (1995). Synchronization between Motor Cortex and Spinal Motoneuronal Pool during the Performance of a Maintained Motor Task in Man. J. Physiol..

[24] Raethjen J., Lindemann M., Dümpelmann M., Wenzelburger R., Stolze H., Pfister G., Elger C., Timmer J., Deuschl G. (2002). Corticomuscular Coherence in the 6-15 Hz Band: Is the Cortex Involved in the Generation of Physiologic Tremor?. Exp. Brain Res..

[25] Salenius S., Hari R. (2003). Synchronous Cortical Oscillatory Activity during Motor Action. Curr. Opin. Neurobiol..

[26] Gross J., Tass P.A., Salenius S., Hari R., Freund H.-J., Schnitzler A. (2000). Cortico-muscular Synchronization during Isometric Muscle Contraction in Humans as Revealed by Magnetoencephalography. J. Physiol..

[27] Marsden J.F. (2000). Coherence between Cerebellar Thalamus, Cortex and Muscle in Man: Cerebellar Thalamus Interactions. Brain.

[28] Ohara S., Mima T., Baba K., Ikeda A., Kunieda T., Matsumoto R., Yamamoto J., Matsuhashi M., Nagamine T., Hirasawa K. (2001). Increased Synchronization of Cortical Oscillatory Activities between Human Supplementary Motor and Primary Sensorimotor Areas during Voluntary Movements. J. Neurosci..

[29] Broniera Junior P., Campos D.P., Lazzaretti A.E., Nohama P., Carvalho A.A., Krueger E., Minhoto Teixeira M.C. (2021). EEG-FES-Force-MMG Closed-Loop Control Systems of a Volunteer with Paraplegia Considering Motor Imagery with Fatigue Recognition and Automatic Shut-Off. Biomed. Signal Processing Control.

[30] Schaefer L.V., Bittmann F.N. (2018). Coherent Behavior of Neuromuscular Oscillations between Isometrically Interacting Subjects: Experimental Study Utilizing Wavelet Coherence Analysis of Mechanomyographic and Mechanotendographic Signals. Sci. Rep..

[31] Schaefer L.V., Torick A.H., Matuschek H., Holschneider M., Bittmann F.N. (2014). Synchronization of Muscular Oscillations Between Two Subjects During Isometric Interaction. Eur. J. Transl. Myol..

[32] Schaefer L.V. (2014). Synchronisationsphänomene myotendinöser Oszillationen interagierender neuromuskulärer Systeme—Mit Betrachtung einer Hypothese bezüglich unterschiedlicher Qualitäten isometrischer Muskelaktion. Ph.D. Thesis.

[33] Pérez A., Carreiras M., Duñabeitia J.A. (2017). Brain-to-Brain Entrainment: EEG Interbrain Synchronization While Speaking and Listening. Sci. Rep..

[34] Liu T., Pelowski M. (2014). A New Research Trend in Social Neuroscience: Towards an Interactive-Brain Neuroscience: Towards an Interactive-Brain Neuroscience. PsyCh J..

[35] Liu N., Mok C., Witt E.E., Pradhan A.H., Chen J.E., Reiss A.L. (2016). NIRS-Based Hyperscanning Reveals Inter-Brain Neural Synchronization during Cooperative Jenga Game with Face-to-Face Communication. Front. Hum. Neurosci..

[36] Kawasaki M., Yamada Y., Ushiku Y., Miyauchi E., Yamaguchi Y. (2013). Inter-Brain Synchronization during Coordination of Speech Rhythm in Human-to-Human Social Interaction. Sci. Rep..

[37] Van Vugt M.K., Pollock J., Johnson B., Gyatso K., Norbu N., Lodroe T., Gyaltsen T., Phuntsok L., Thakchoe J., Khechok J. (2020). Inter-Brain Synchronization in the Practice of Tibetan Monastic Debate. Mindfulness.

[38] Fishburn F.A., Murty V.P., Hlutkowsky C.O., MacGillivray C.E., Bemis L.M., Murphy M.E., Huppert T.J., Perlman S.B. (2018). Putting Our Heads Together: Interpersonal Neural Synchronization as a Biological Mechanism for Shared Intentionality. Soc. Cogn. Affect. Neurosci..

[39] Nozawa T., Sakaki K., Ikeda S., Jeong H., Yamazaki S., Kawata K.H.D.S., Kawata N.Y.D.S., Sasaki Y., Kulason K., Hirano K. (2019). Prior Physical Synchrony Enhances Rapport and Inter-Brain Synchronization during Subsequent Educational Communication. Sci. Rep..

[40] Dumas G., Nadel J., Soussignan R., Martinerie J., Garnero L. (2010). Inter-Brain Synchronization during Social Interaction. PLoS ONE.

[41] Balconi M., Fronda G. (2020). The Use of Hyperscanning to Investigate the Role of Social, Affective, and Informative Gestures in Non-Verbal Communication. Electrophysiological (EEG) and Inter-Brain Connectivity Evidence. Brain Sci..

[42] Balconi M., Fronda G. (2021). Intra-Brain Connectivity vs. Inter-Brain Connectivity in Gestures Reproduction: What Relationship?. Brain Sci..

[43] Takeuchi N., Izumi S.-I. (2021). Motor Learning Based on Oscillatory Brain Activity Using Transcranial Alternating Current Stimulation: A Review. Brain Sci..

[44] Müller V., Sänger J., Lindenberger U. (2013). Intra- and Inter-Brain Synchronization during Musical Improvisation on the Guitar. PLoS ONE.

[45] Hennig H. (2014). Synchronization in Human Musical Rhythms and Mutually Interacting Complex Systems. Proc. Natl. Acad. Sci. USA.

[46] Lindenberger U., Li S.-C., Gruber W., Müller V. (2009). Brains Swinging in Concert: Cortical Phase Synchronization While Playing Guitar. BMC Neurosci..

[47] Novembre G., Iannetti G.D. (2021). Hyperscanning Alone Cannot Prove Causality. Multibrain Stimulation Can. Trends Cogn. Sci..

[48] Gvirts Provolovski H.Z., Perlmutter R. (2021). How Can We Prove the Causality of Interbrain Synchronization?. Front. Hum. Neurosci..

[49] Hasson U., Frith C.D. (2016). Mirroring and beyond: Coupled Dynamics as a Generalized Framework for Modelling Social Interactions. Phil. Trans. R. Soc. B.

[50] Bittmann F.N., Dech S., Aehle M., Schaefer L.V. (2020). Manual Muscle Testing—Force Profiles and Their Reproducibility. Diagnostics.

[51] Schaefer L.V., Dech S., Aehle M., Bittmann F.N. (2021). Disgusting Odours Affect the Characteristics of the Adaptive Force in Contrast to Neutral and Pleasant Odours. Sci. Rep..

[52] Schaefer L.V., Dech S., Bittmann F.N. (2021). Adaptive Force and Emotionally Related Imaginations—Preliminary Results Suggest a Reduction of the Maximal Holding Capacity as Reaction to Disgusting Food Imagination. Heliyon.

[53] Schaefer L.V., Dech S., Wolff L.L., Bittmann F.N. (2022). Influence of Emotionally Affective Imaginations on the Adaptive Force in Young Women: Unpleasant Imaginations Reduce the Holding Capacity of Muscles. Res. Sq..

[54] Conable K.M., Rosner A.L. (2011). A Narrative Review of Manual Muscle Testing and Implications for Muscle Testing Research. J. Chiropr. Med..

[55] Hunter S.K., Ryan D.L., Ortega J.D., Enoka R.M. (2002). Task Differences with the Same Load Torque Alter the Endurance Time of Submaximal Fatiguing Contractions in Humans. J. Neurophysiol..

[56] Rudroff T., Kalliokoski K.K., Block D.E., Gould J.R., Klingensmith W.C., Enoka R.M. (2013). PET/CT Imaging of Age- and Task-Associated Differences in Muscle Activity during Fatiguing Contractions. J. Appl. Physiol..

[57] Rudroff T., Barry B.K., Stone A.L., Barry C.J., Enoka R.M. (2007). Accessory Muscle Activity Contributes to the Variation in Time to Task Failure for Different Arm Postures and Loads. J. Appl. Physiol..

[58] Rudroff T., Justice J.N., Holmes M.R., Matthews S.D., Enoka R.M. (2011). Muscle Activity and Time to Task Failure Differ with Load Compliance and Target Force for Elbow Flexor Muscles. J. Appl. Physiol..

[59] Semmler J.G., Kornatz K.W., Dinenno D.V., Zhou S., Enoka R.M. (2002). Motor Unit Synchronisation Is Enhanced during Slow Lengthening Contractions of a Hand Muscle. J. Physiol..

[60] Schaefer L.V., Bittmann F.N. (2021). Paired personal interaction reveals objective differences between pushing and holding isometric muscle action. PLoS ONE.

[61] Schaefer L.V., Bittmann F.N. (2017). Are There Two Forms of Isometric Muscle Action? Results of the Experimental Study Support a Distinction between a Holding and a Pushing Isometric Muscle Function. BMC Sports Sci. Med. Rehabil..

[62] Garner J.C., Blackburn T., Weimar W., Campbell B. (2008). Comparison of Electromyographic Activity during Eccentrically versus Concentrically Loaded Isometric Contractions. J. Electromyogr. Kinesiol..

[63] Schaefer L.V., Bittmann F.N. (2021). Mechanotendography: Description and Evaluation of a Novel Method for Investigating the Physiological Mechanical Oscillations of Tendons Using a Piezo-Based Measurement System. Eur. J. Transl. Myol..

[64] Zion-Golumbic E. (2007). What Is EEG?. https://www.mada.org.il/brain/articles/faces-e.pdf.

[65] Schlögl A., Slater M., Pfurtscheller G. Presence research and EEG 2002. Proceedings of the 5th International Workshop on Presence.

[66] Rajya L.M., Prasad T.V., Prakash V.C. (2014). Survey on EEG Signal Processing Methods. Int. J. Adv. Res. Comput. Sci. Softw. Eng..

[67] Pontifex M.B., Gwizdala K.L., Parks A.C., Billinger M., Brunner C. (2017). Variability of ICA Decomposition May Impact EEG Signals When Used to Remove Eyeblink Artifacts: ICA Variability. Psychophysiology.

[68] Meiron O., Gale R., Namestnic J., Bennet-Back O., Gebodh N., Esmaeilpour Z., Mandzhiyev V., Bikson M. (2019). Antiepileptic Effects of a Novel Non-Invasive Neuromodulation Treatment in a Subject with Early-Onset Epileptic Encephalopathy: Case Report With 20 Sessions of HD-TDCS Intervention. Front. Neurosci..

[69] Alotaiby T., El-Samie F.E.A., Alshebeili S.A., Ahmad I. (2015). A Review of Channel Selection Algorithms for EEG Signal Processing. EURASIP J. Adv. Signal. Process..

[70] Ernst K. (2020). Quantitative EEG-Auswertung Mittels Kohärenz- und Power-Analyse bei Fokalen Epilepsien. Ph.D. Thesis.

[71] Schaefli B., Maraun D., Holschneider M. (2007). What Drives High Flow Events in the Swiss Alps? Recent Developments in Wavelet Spectral Analysis and Their Application to Hydrology. Adv. Water Resour..

[72] Holschneider M. (1995). Wavelets. An Analysis Tool.

[73] Maraun D., Kurths J., Holschneider M. (2007). Nonstationary Gaussian Processes in Wavelet Domain: Synthesis, Estimation, and Significance Testing. Phys. Rev. E.

[74] Pfurtscheller G., Lopes Da Silva F.H. (1999). Event-Related EEG/MEG Synchronization and Desynchronization: Basic Principles. Clin. Neurophysiol..

[75] Cohen J. (1992). A Power Primer. Psychol. Bull..

[76] Sullivan G.M., Feinn R. (2012). Using effect size—Or why the *p* value is not enough. J. Grad. Med. Educ..

[77] Rothman K.J. (1990). No Adjustments Are Needed for Multiple Comparisons. Epidemiology.

[78] Bender R., Lange S., Ziegler A. (2002). Multiples Testen. DMW Dtsch. Med. Wochenschr..

[79] O’Brien P.C. (1983). The Appropriateness of Analysis of Variance and Multiple-Comparison Procedures. Biometrics.

[80] Babiloni F., Cincotti F., Carducci F., Rossini P.M., Babiloni C. (2001). Spatial Enhancement of EEG Data by Surface Laplacian Estimation: The Use of Magnetic Resonance Imaging-Based Head Models. Clin. Neurophysiol..

[81] Albus J.S. (1971). A Theory of Cerebellar Function. Math. Biosci..

[82] Ashe J., Bushara K., Merchant H., De Lafuente V. (2014). The Olivo-Cerebellar System as a Neural Clock. Neurobiology of Interval Timing.

[83] Lawrenson C., Bares M., Kamondi A., Kovács A., Lumb B., Apps R., Filip P., Manto M. (2018). The Mystery of the Cerebellum: Clues from Experimental and Clinical Observations. Cerebellum Ataxias.

[84] Shadmehr R., Smith M.A., Krakauer J.W. (2010). Error Correction, Sensory Prediction, and Adaptation in Motor Control. Annu. Rev. Neurosci..

[85] Doya K. (2000). Complementary Roles of Basal Ganglia and Cerebellum in Learning and Motor Control. Curr. Opin. Neurobiol..

[86] Bueti D., Walsh V., Frith C., Rees G. (2008). Different Brain Circuits Underlie Motor and Perceptual Representations of Temporal Intervals. J. Cogn. Neurosci..

[87] Rao S.M., Harrington D.L., Haaland K.Y., Bobholz J.A., Cox R.W., Binder J.R. (1997). Distributed Neural Systems Underlying the Timing of Movements. J. Neurosci..

[88] Lewis P.A., Miall R.C. (2003). Brain Activation Patterns during Measurement of Sub- and Supra-Second Intervals. Neuropsychologia.

[89] Huggenberger S., Moser N., Schröder H., Cozzi B., Granato A., Merighi A. (2019). Neuroanatomie Des. Menschen: Mit 202 Größtenteils Farbigen Abbildungen.

[90] Vogt B.A., Finch D.M., Olson C.R. (1992). Functional Heterogeneity in Cingulate Cortex: The Anterior Executive and Posterior Evaluative Regions. Cereb. Cortex.

[91] Groenewegen H.J. (2003). The Basal Ganglia and Motor Control. Neural Plast..

[92] Gerfen C.R., Wilson C.J. (1996). Chapter II the Basal Ganglia. Handbook of Chemical Neuroanatomy.

[93] Morecraft R.J., Tanjii J. (2009). Cingulofrontal Interactions and the Cingulate Motor Areas. Cingulate Neurobiology and Disease.

[94] Welsh J.P., Lang E.J., Suglhara I., Llinás R. (1995). Dynamic Organization of Motor Control within the Olivocerebellar System. Nature.

[95] Sherman S. (2006). Thalamus. Scholarpedia.

[96] Vogt B.A., Nimchinsky E.A., Vogt L.J., Hof P.R. (1995). Human Cingulate Cortex: Surface Features, Flat Maps, and Cytoarchitecture. J. Comp. Neurol..

[97] Ivry R.B. (1996). The Representation of Temporal Information in Perception and Motor Control. Curr. Opin. Neurobiol..

[98] Schaefer L.V., Löffler N., Klein J., Bittmann F.N. (2021). Mechanomyography and Acceleration Show Interlimb Asymmetries in Parkinson Patients without Tremor Compared to Controls during a Unilateral Motor Task. Sci. Rep..

[99] Schaefer L.V., Bittmann F.N. (2020). Parkinson Patients without Tremor Show Changed Patterns of Mechanical Muscle Oscillations during a Specific Bilateral Motor Task Compared to Controls. Sci. Rep..

[100] Stancák A., Pfurtscheller G. (1996). Event-Related Desynchronisation of Central Beta-Rhythms during Brisk and Slow Self-Paced Finger Movements of Dominant and Nondominant Hand. Cogn. Brain Res..

[101] Enoka R.M. (1996). Eccentric Contractions Require Unique Activation Strategies by the Nervous System. J. Appl. Physiol..

[102] Duchateau J., Baudry S. (2014). Insights into the Neural Control of Eccentric Contractions. J. Appl. Physiol..

[103] Duchateau J., Enoka R.M. (2016). Neural Control of Lengthening Contractions. J. Exp. Biol..

[104] Duchateau J., Enoka R.M. (2008). Neural Control of Shortening and Lengthening Contractions: Influence of Task Constraints: Shortening and Lengthening Contractions. J. Physiol..

[105] O’Halloran C.J., Kinsella G.J., Storey E. (2012). The Cerebellum and Neuropsychological Functioning: A Critical Review. J. Clin. Exp. Neuropsychol..

[106] Schmahmann J.D., Caplan D. (2006). Cognition, Emotion and the Cerebellum. Brain.

[107] Sänger J., Müller V., Lindenberger U. (2012). Intra- and Interbrain Synchronization and Network Properties When Playing Guitar in Duets. Front. Hum. Neurosci..

