# Supplementary material for: Case Study: Intra- and Interpersonal Coherence of Muscle and Brain Activity of Two Coupled Persons during Pushing and Holding Isometric Muscle Action
Source: Brain Sci. 2022 May 29;12(6):703. doi: 10.3390/brainsci12060703 (PMC9221481; doi:10.3390/brainsci12060703)
Supplement: Supplementary file 1 [file brainsci-12-00703-s001.zip › brainsci-1673921-supplementary.pdf]

brain sciences

MDPI

Supplementary material

Article

Case study: intra- and interpersonal coherence of muscle and brain activity of two coupled persons during pushing and holding isometric muscle action

(Schaefer LV, Bittmann FN)

Content

Figure S1. Exemplary signals.. 2

Table S1. Inter-brain Sum5PaD motor vs. non-motor tasks. 3

Table S2. Sum5PaD of force and ACC to MMGs and EEG real vs. random pairs................ 3

Table S3. Intra- and interpersonal Sum5PaD of coupled real vs. single-MVIC pairs........... 4

Table S4. Intrapersonal muscle-brain coherence HIMA vs. PIMA.. 5

Table S5. Interpersonal muscle-brain coherence HIMA vs. PIMA.. 5

Figure S1. *Exemplary signals*

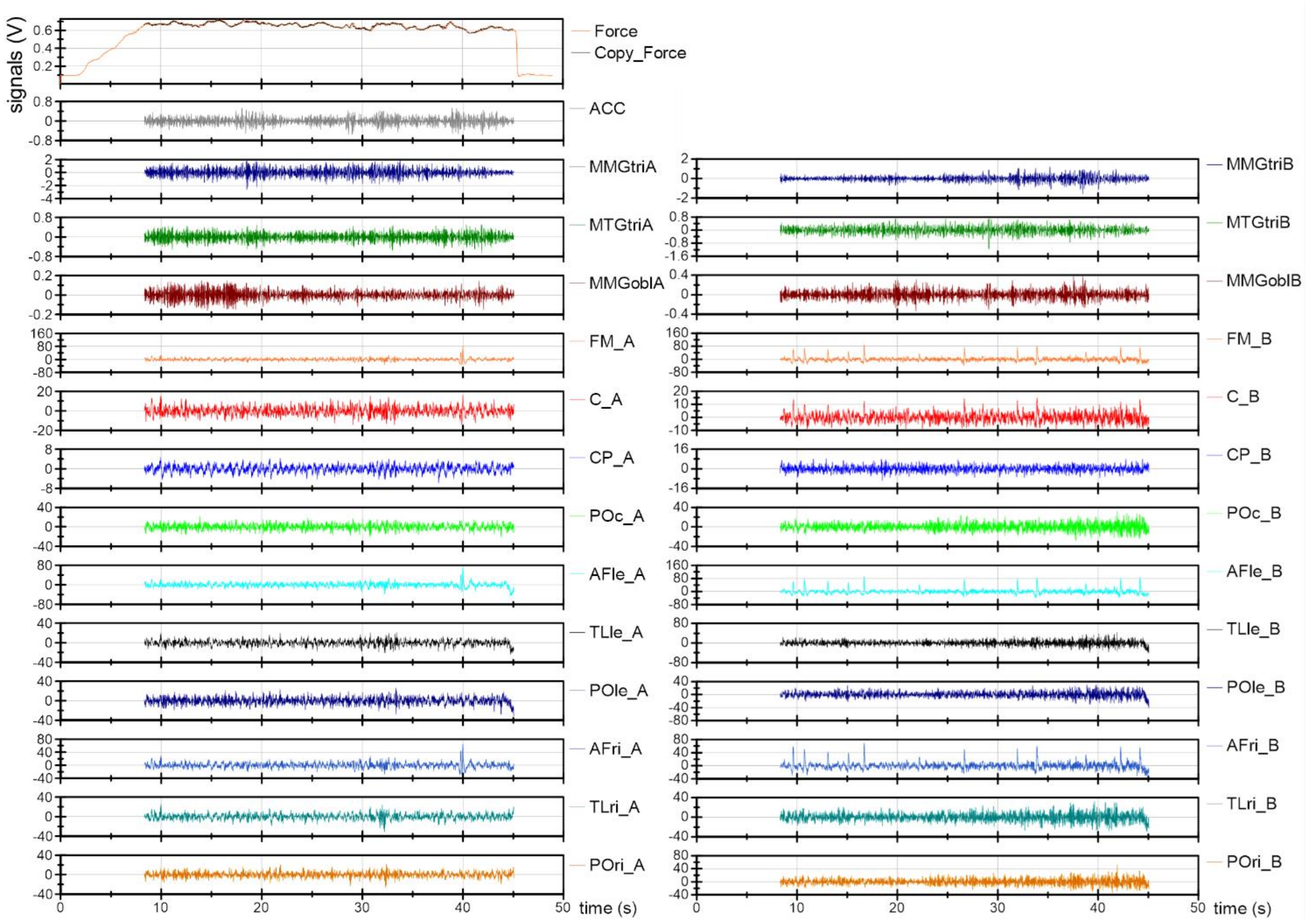

Figure S1. Exemplary signals. Displayed are the force, ACC, MMG/MTG and averaged EEG signals (sub-regions) of partner A (left) and B (right) during the isometric plateau of one trial (A-PIMA_B-HIMA_1). Signals are filtered and down-sampled.

Table S1. *Inter-brain Sum5PaD motor vs. non-motor tasks.*

Arithmetic means (M), standard deviations (SD), coefficient of variation (CV), t values of paired t-test or z values of Wilcoxon test, degrees of freedom (df), significance p and effect sizes Cohen's d_z or Pearson's r are given comparing inter-brain coherence of trials during coupled isometric muscle action (AB_IMA) vs. measurement without motor task but with opened eyes (OpEy).

Sensor pair	Mode	n	M (%)	SD	CV	t / z	df	p	d_z / r
EEGcen	AB_IMA	10	19.082	4.678	0.245	-2.497	-	**0.013***	0.790
	OpEy		9.654	5.228	0.542				
EEGle	AB_IMA	6	21.94	4.974	0.227	-2.201	-	**0.028***	0.782
	OpEy		12.660	3.170	0.250				
EEGri	AB_IMA	6	20.556	3.481	0.169	5.017	5	**0.004**	2.048
	OpEy		10.194	5.552	0.545				
EEGcen vs. EEGle	AB_IMA	12	20.874	4.645	0.223	3.500	11	**0.005**	1.010
	OpEy		13.199	5.436	0.412				
EEGcen vs. EEGri	AB_IMA	12	19.603	3.126	0.159	3.838	11	**0.003**	1.108
	OpEy		11.145	6.085	0.546				
EEGle vs. EEGri	AB_IMA	9	21.32	3.573	0.168	5.253	8	**0.001**	1.751
	OpEy		13.597	5.609	0.413				

*Wilcoxon test. Significant results are written in bold.

Table S2. *Sum5PaD of force and ACC to MMGs and EEG real vs. random pairs.*

Arithmetic means (M), standard deviations (SD), coefficient of variation (CV), t values of paired t-test or z value of Wilcoxon test, degrees of freedom (df), significance p and effect size Cohen's d_z or Pearson's r of the comparisons between real (AB_IMA) and random pairs (rand) of the parameter Sum5PaD of force and ACC vs. MMGs and EEGs are given.

Sensor pair	Mode	n	M (%)	SD	CV	t / z	df	p	d_z / r
Force vs. MMGs	AB_IMA	6	89.820	26.470	0.295	6.035	5	**0.002**	2.464
	rand		17.690	14.045	0.794				
Force vs. EEGcen	AB_IMA	8	41.379	12.682	0.306	5.640	7	**0.001**	1.994
	rand		20.948	10.159	0.485				
Force vs. EEGle	AB_IMA	6	43.746	17.912	0.409	-1.992	-	**0.046***	0.813
	rand		18.233	15.781	0.866				
Force vs. EEGri	AB_IMA	6	44.754	5.823	0.130	3.530	5	**0.017**	1.441
	rand		22.406	13.880	0.619				
ACC vs. MMGs	AB_IMA	6	105.825	23.729	0.224	9.006	5	**< 0.001**	3.676
	rand		22.665	12.044	0.531				
ACC vs. EEGcen	AB_IMA	8	46.744	18.113	0.387	4.868	7	**0.002**	1.721
	rand		13.830	13.084	0.946				
ACC vs. EEGle	AB_IMA	6	53.871	28.217	0.524	4.086	5	**0.009**	1.668
	rand		14.428	13.547	0.939				
ACC vs. EEGri	AB_IMA	6	48.229	10.014	0.208	3.203	5	**0.024**	1.308
	rand		29.294	12.948	0.442				

* Wilcoxon test, effect size is given by Pearson's r. Significant results are written in bold.

Table S3. *Intra- and interpersonal Sum5PaD of coupled real vs. single-MVIC pairs.*

Arithmetic means (M), standard deviations (SD), coefficient of variation (CV), t values of paired t-test or z values of Wilcoxon test, degrees of freedom (df), significance p and effect size Cohen's dz of the signal pairs during the HIMA-PIMA trials of the coupled partners (AB_IMA) vs. MVIC trials for all regions are given for intrapersonal (above) and interpersonal (below) considerations. For the comparison of the sub-regions of the same region only MMGs, EEGcen, EEGle and EEGri is noted.

Sensor pair	Mode	n	M (%)	SD	CV	t / z	df	p	d_z / r
**Intrapersonal coherence**									
MMGs	AB_IMA	3	97.097	4.895	0.050	2.973	2	0.065	2.146
	MVIC		62.845	11.150	0.177				
MMGs vs. EEGcen	AB_IMA	12	26.164	9.840	0.376	1.334	-	0.182*	-
	MVIC		41.935	27.238	0.650				
MMGs vs. EEGle	AB_IMA	9	33.735	9.623	0.285	-1.436	8	0.189	0.479
	MVIC		44.092	22.214	0.504				
MMGs vs. EEGri	AB_IMA	9	28.557	6.406	0.224	-1.869	8	0.099	0.623
	MVIC		43.916	24.555	0.559				
EEGcen	AB_IMA	6	89.240	14.730	0.165	1.833	5	0.126	0.748
	MVIC		66.811	32.648	0.489				
EEGle	AB_IMA	3	90.717	34.711	0.383	0.696	2	0.588	0.402
	MVIC		82.536	55.039	0.667				
EEGri	AB_IMA	3	99.323	24.465	0.246	4.291	2	0.050	2.477
	MVIC		88.010	26.336	0.299				
EEGcen vs. EEGle	AB_IMA	12	90.205	12.243	0.136	3.259	11	**0.008**	0.941
	MVIC		78.925	14.149	0.179				
EEGcen vs. EEGri	AB_IMA	12	89.883	10.240	0.114	3.421	11	**0.006**	0.987
	MVIC		73.361	20.196	0.275				
EEGle vs. EEGri	AB_IMA	9	102.419	16.432	0.160	1.506	8	0.171	0.502
	MVIC		89.631	20.814	0.232				
**Interpersonal coherence**									
MMGs	AB_IMA	6	62.450	23.429	0.375	1.721	5	0.146	0.702
	MVIC		37.037	14.174	0.383				
MMGs vs. EEGcen	AB_IMA	12	55.472	15.429	0.278	5.784	11	**< 0.001**	1.67
	MVIC		29.969	17.85	0.596				
MMGs vs. EEGle	AB_IMA	9	63.187	15.588	0.247	4.849	8	**0.001**	1.616
	MVIC		31.747	11.084	0.349				
MMGs vs. EEGri	AB_IMA	9	61.155	11.564	0.189	5.456	8	**0.001**	1.819
	MVIC		26.071	18.73	0.718				
EEGcen	AB_IMA	10	19.082	4.678	0.245	-0.693	9	0.506	0.219
	MVIC		22.622	14.503	0.641				
EEGle	AB_IMA	6	21.94	4.974	0.227	1.301	5	0.250	0.531
	MVIC		17.488	10.785	0.617				
EEGri	AB_IMA	6	20.556	3.481	0.169	1.921	5	0.113	0.784
	MVIC		13.096	10.178	0.777				
EEGcen vs. EEGle	AB_IMA	12	20.874	4.645	0.223	0.638	11	0.537	0.184
	MVIC		18.888	9.898	0.524				
EEGcen vs. EEGri	AB_IMA	12	19.603	3.126	0.159	-0.846	11	0.416	0.244
	MVIC		21.738	8.594	0.395				
EEGle vs. EEGri	AB_IMA	9	21.32	3.573	0.168	0.429	8	0.679	0.143
	MVIC		19.762	10.998	0.557				

*Wilcoxon test. Significant results are written in bold.

Table S4. *Intrapersonal muscle-brain coherence HIMA vs. PIMA.*

Arithmetic means (M), standard deviations (SD), coefficient of variation (CV), F values of RM ANOVA or z values of Friedman test, degrees of freedom (df), significance p and effect size eta-squared of the muscle-brain coherence (Sum5PaD) comparing real coupled trials during HIMA vs. PIMA and vs. MVIC trials (PIMA) are given for intrapersonal considerations.

**Sensor pair**	**Mode**	**n**	**M (%)**	**SD**	**CV**	**F / z**	**df**	**p**	**η^2**
**Intrapersonal coherence**									
MMGs vs. EEGcen	HIMA	24	26.085	9.454	0.362	4.083*	-	0.130	-
	PIMA		26.243	17.400	0.663				
	MVIC		41.935	31.336	0.747				
MMGs vs. EEGle	HIMA	18	36.306	12.626	0.348	2.599[a]	1.4, 23.3	0.111	-
	PIMA		31.165	15.658	0.502				
	MVIC		44.092	29.368	0.666				
MMGs vs. EEGri	HIMA	18	30.120	9.550	0.317	5.367[a]	1.6, 19.8	**0.027**	0.24
	PIMA		26.994	7.805	0.289				
	MVIC		43.916	26.254	0.598				

*Friedman test. [a]Greenhouse-Geisser correction. Significant results are written in bold.

Table S5. *Interpersonal muscle-brain coherence HIMA vs. PIMA.*

Arithmetic means (M), standard deviations (SD), coefficient of variation (CV), values of paired t test, degrees of freedom (df), significance p and effect size Cohen's d_z of the interpersonal muscle-brain coherence (Sum5PaD) comparing HIMA vs. PIMA of real coupled trials are given.

**Sensor pair**	**Mode**	**n**	**M (%)**	**SD**	**CV**	**t**	**df**	**p**	**d_z**
**Interpersonal coherence**									
MMGs vs. EEGcen	HIMA	8	58.568	16.869	0.288	-2.406	7	**0.047**	0.673
	PIMA		52.376	17.584	0.336				
	MVIC		69.240	20.231	0.292				
MMGs vs. EEGle	HIMA	6	57.135	19.789	0.346	-4.429	5	**0.007**	0.893
	PIMA		64.165	18.915	0.295				
	MVIC		58.145	17.430	0.300				
MMGs vs. EEGri	HIMA	6	58.568	16.869	0.288	-1.783	5	0.135	-
	PIMA		52.376	17.584	0.336				
	MVIC		69.240	20.231	0.292				

Significant results are written in bold.
